# Nearly Periodic Maps and Geometric Integration of Noncanonical Hamiltonian Systems

**DOI:** 10.1007/s00332-023-09891-4

**Published:** 2023-02-25

**Authors:** J. W. Burby, E. Hirvijoki, M. Leok

**Affiliations:** 1 Los Alamos National Laboratory, Los Alamos, NM 87545 USA; 2 Department of Mechanical Engineering, Aalto University, PO Box 14400, 00076 Espoo, Finland; 3 Department of Mathematics, University of California, San Diego, 9500 Gilman Drive, La Jolla, CA 92093-0112 USA

## Abstract

M. Kruskal showed that each continuous-time nearly periodic dynamical system admits a formal *U*(1)-symmetry, generated by the so-called roto-rate. When the nearly periodic system is also Hamiltonian, Noether’s theorem implies the existence of a corresponding adiabatic invariant. We develop a discrete-time analog of Kruskal’s theory. Nearly periodic maps are defined as parameter-dependent diffeomorphisms that limit to rotations along a *U*(1)-action. When the limiting rotation is non-resonant, these maps admit formal *U*(1)-symmetries to all orders in perturbation theory. For Hamiltonian nearly periodic maps on exact presymplectic manifolds, we prove that the formal *U*(1)-symmetry gives rise to a discrete-time adiabatic invariant using a discrete-time extension of Noether’s theorem. When the unperturbed *U*(1)-orbits are contractible, we also find a discrete-time adiabatic invariant for mappings that are merely presymplectic, rather than Hamiltonian. As an application of the theory, we use it to develop a novel technique for geometric integration of non-canonical Hamiltonian systems on exact symplectic manifolds.

## Introduction

A continuous-time dynamical system with vector parameter $\gamma$ is nearly periodic if all of its trajectories are periodic with nowhere-vanishing angular frequency in the limit $\gamma \rightarrow 0$. Examples from physics include charged particle dynamics in a strong magnetic field, the weakly relativistic Dirac equation, and any mechanical system subject to a high-frequency, time-periodic force. In the broader context of multi-scale dynamical systems, nearly periodic systems play a special role because they display perhaps the simplest possible non-dissipative short-timescale dynamics. They therefore provide a useful proving ground for analytical and numerical methods aimed at more complex multi-scale models.

In a seminal paper (Kruskal 1962), Kruskal deduced the basic asymptotic properties of continuous-time nearly periodic systems. In general, each such system admits a formal *U*(1)-Lie symmetry whose infinitesimal generator $R_\gamma$ is known as the roto-rate. In the Hamiltonian setting, existence of the roto-rate implies existence of an all-orders adiabatic invariant $\mu _\gamma$ by way of Noether’s theorem. General expressions for $\mu _\gamma$ may be found in Burby and Squire (2020). Recently (Burby and Hirvijoki 2021), we extended Kruskal’s analysis by proving that the (formal) set of fixed points for the roto-rate is an elliptic almost invariant slow manifold. Moreover, in the Hamiltonian case, we demonstrated that normal stability of the slow manifold is mediated by Kruskal’s adiabatic invariant.

The purpose of this article is to introduce discrete-time analogs of continuous-time nearly periodic systems that we call nearly periodic maps. These objects can be motivated as follows. A nearly periodic system characteristically displays limiting short-timescale dynamics that ergodically covers circles in phase space. This ergodicity is ultimately what gives rise to Kruskal’s roto-rate and, in the presence of Hamiltonian structure, adiabatic invariance. It is therefore sensible to regard parameter-dependent maps whose limiting iterations ergodically cover circles as discrete-time analogs of nearly periodic systems. Ergodicity requires that the rotation angle associated with each circle be an irrational multiple of $2\pi$. In principle, these rotation angles could vary from circle to circle, but smoothness removes this freedom and imposes a common rotation angle across circles. Nearly periodic maps are defined by limiting iterations that rotate a family of circles foliating phase space by a common rotation angle. Such a map is resonant or non-resonant when the rotation angle is a rational or irrational multiple of $2\pi$, respectively. The preceding remarks suggest that non-resonant nearly periodic maps should share important features with continuous-time nearly periodic systems.

We will show that non-resonant nearly periodic maps always admit formal *U*(1)-symmetries by modifying Kruskal’s construction of a normal form for the roto-rate. Thus, non-resonant nearly periodic maps formally reduce to mappings on the space of *U*(1)-orbits, corresponding to elimination of a single dimension in phase space. In the Hamiltonian setting, we will establish a discrete-time analog of Noether’s theorem that will allow us to construct all-orders adiabatic invariants for non-resonant nearly periodic maps. In contrast to the continuous-time case, there may be topological obstructions to the Noether theorem-based construction. Nevertheless, assuming (a) existence of a fixed point for formal *U*(1)-symmetry, or (b) existence of a time-dependent Hamiltonian suspension for the nearly periodic map ( a time-dependent Hamiltonian flow that interpolates between the identity and the nearly periodic map), these topological obstructions disappear. When an adiabatic invariant does exist, the phase-space dimension is formally reduced by two instead of one. On the slow manifold, corresponding to vanishing of the adiabatic invariant, the reduction in dimensionality may be even more dramatic.

We anticipate that non-resonant nearly periodic maps will have important applications to numerical integration of nearly periodic systems. While development of integrators for such systems is straightforward when the numerical timestep *h* resolves the short-timescale dynamics, considerably more care is required when “stepping over” the period of limiting oscillations. One approach would be to design an integrator on the unreduced space that is constrained to be a non-resonant nearly periodic map. Although such an integrator would not accurately resolve the phase of short-scale oscillations when taking large timesteps, it would automatically possess an all-orders reduction to the space of *U*(1)-orbits. By designing the reduced map to discretize the continuous-time reduced dynamics, the slow component of the continuous-time dynamics could be accurately resolved without directly simulating the reduced dynamical variables. This opens the door to a type of asymptotic-preserving integrator capable of seamlessly transitioning between large- and small-timestep regimes, generalizing those proposed in Ricketson and Chacón (2020), Xiao and Qin (2021) for magnetized charged particle dynamics. Moreover, in the Hamiltonian case, the integrator would automatically enjoy an all-orders adiabatic invariant close to the continuous-time invariant. Such a capability would complement previous results on short-timestep adiabatic invariants for variational integrators (Hairer and Lubich 2020). We provide a proof-of-principle demonstration of these ideas in Sect. 5.1

Aside from serving as integrators for nearly periodic systems, nearly periodic maps may also be used as tools for structure-preserving simulation of general Hamiltonian systems on exact symplectic manifolds. (See Abraham and Marsden 2008; Marsden and Ratiu 1999 for the foundations of Hamiltonian mechanics on symplectic manifolds.) The basic idea is to first embed the original Hamiltonian system as an approximate invariant manifold inside of a larger nearly periodic Hamiltonian system, as discussed in Burby and Hirvijoki (2021). Then, it is possible to construct a symplectic nearly periodic map that integrates the larger system while preserving the approximate invariant manifold. Discrete-time adiabatic invariance ensures that the approximate invariant manifold enjoys long-term normal stability, which is tantamount to the integrator providing a persistent approximation of the original system’s dynamics. We describe and analyze this construction in Sect. 4.2. In Sect. 5.2, we apply the general theory to the non-canonical Hamiltonian dynamics of a charged particle’s guiding center (Northrop 1963; Littlejohn 1981, 1983) in a magnetic field of the form ${\varvec{B}} = B(x,y)\,{\varvec{e}}_z$ (Littlejohn 1979).

The remainder of this article is organized as follows. After providing a brief non-technical overview, we review Kruskal’s theory of nearly periodic systems using modern terminology in Sect. 3. Then, we develop the general theory of nearly periodic maps in Sect. 4, including their special properties in the symplectic case, and their ability to serve as geometric integrators for Hamiltonian systems on exact symplectic manifolds. Wherever possible, proofs of general properties of nearly periodic maps parallel Kruskal’s arguments from the continuous-time setting. Section 5 contains a pair of interesting applications of nearly periodic map technology. Finally, Sect. 6 provides additional review and context for this work.

### Notational Conventions

In this article, smooth shall always mean $C^\infty$, and $\Gamma$ will always denote a vector space. We reserve the symbol *M* for a smooth manifold equipped with a smooth auxiliary Riemannian metric *g*. We say $f_\gamma :M_1\rightarrow M_2$, $\gamma \in \Gamma$, is a smooth $\gamma$-dependent mapping between manifolds $M_1,M_2$ when the mapping $M_1\times {\mathbb {R}}\rightarrow M_2:(m,\gamma )\mapsto f_\gamma (m)$ is smooth. Similarly, ${\varvec{T}}_\gamma$ is a smooth $\gamma$-dependent tensor field on *M* when (a) ${\varvec{T}}_\gamma (m)$ is an element of the tensor algebra ${\mathcal {T}}_m(M)$ at *m* for each $m\in M$ and $\gamma \in \Gamma$, and (b) ${\varvec{T}}_\gamma$ is a smooth $\gamma$-dependent mapping between the manifolds *M* and ${\mathcal {T}}(M)=\bigcup _{m\in M}{\mathcal {T}}_m(M)$.

The symbol $X_\gamma$ will always denote a smooth $\gamma$-dependent vector field on *M*. If ${\varvec{T}}_\gamma$ is a smooth $\gamma$-dependent section of either $TM\otimes TM$ or $T^*M\otimes T^*M$, then $\widehat{{\varvec{T}}}_\gamma$ is the corresponding smooth $\gamma$-dependent bundle map $T^*M\rightarrow TM:\alpha \mapsto \iota _\alpha {\varvec{T}}_\gamma$, or $TM\rightarrow T^*M:X\mapsto \iota _X{\varvec{T}}_\gamma$, respectively. Note that if $\Omega$ is a symplectic form on *M* with associated Poisson bivector ${\mathcal {J}}$ then ${\widehat{\Omega }}^{-1} = -\widehat{{\mathcal {J}}}$. Finally, we introduce the following definition to address presymplectic forms that depend on a parameter.

#### Definition 1

Let $\Gamma \ni \gamma$ be a finite-dimensional vector space. A $\gamma$-**dependent presymplectic manifold** is a manifold *M* equipped with a smooth $\gamma$-dependent 2-form $\Omega _\gamma$ such that ${\textbf{d}}\Omega _\gamma = 0$ for each $\gamma \in \Gamma$. We say $(M,\Omega _\gamma )$ is **exact** when there is a smooth $\gamma$-dependent 1-form $\vartheta _\gamma$ such that $\Omega _\gamma = -{\textbf{d}}\vartheta _\gamma$.

## Overview

This article contains a pair of interrelated contributions, an analog of Kruskal’s nearly periodic system theory in discrete time, and an application of the new theory to structure-preserving integration of non-canonical Hamiltonian systems. Each contribution requires understanding a fair amount of technical background material to fully digest. This section therefore aims to present a (mostly) non-technical synopsis of our work. Readers interested in proceeding directly to the technical content should start with Sect. 3.

Kruskal’s theory Kruskal (1962) represented an outgrowth of vigorous investigations into charged particle motion in strong magnetic fields as part of Project Matterhorn, the first serious attempt by the US government to develop nuclear fusion for peaceful energy production. Such charged particles execute rapid rotation around magnetic field lines superposed on a much slower drift motion. Moreover, the magnetic flux that threads their tight helical trajectories remains approximately constant in time over large time intervals, almost as if each particle behaved as a superconducting ring of current. Kruskal recognized that this problem comprised just one example of a rich class of multi-scale dynamical systems for which much could be said using analytical methods. These systems, now called nearly periodic, exhibit two time scales, a short one on which every trajectory is periodic, and a much longer one associated with slow drifting motion. Kruskal also recognized that the magnetic flux invariant from charged particle theory generalizes to nearly periodic systems *provided* the nearly periodic system admits Hamiltonian structure.

Nearly periodic systems bear similarities with the nearly integrable systems addressed by Kolmogorov–Arnol’d–Moser (KAM) theory. They depend on a small parameter $\epsilon$ that measures a degree of timescale separation. When $\epsilon \rightarrow 0$, all trajectories become very simple—even explicitly integrable in the right coordinate system. In fact, nearly periodic systems may be viewed as special examples of nearly integrable systems. However, where nearly integrable systems generically exhibit resonances nearly periodic systems never do. The limiting dynamics for a nearly integrable Hamiltonian system comprise quasiperiodic motion on invariant Lagrangian tori in phase space characterized by several fast phases. On many of these tori, the fast frequencies exhibit integer relationships that notoriously cause perturbation series to break down. But for a nearly periodic system the tori are one-dimensional, making resonance impossible. Kruskal exploited this lack of obstruction to perturbative methods to prove a remarkable result: every nearly periodic system admits a unique hidden continuous symmetry at the level of perturbation theory. While Kruskal did not prove that the series defining his symmetry converge or represent a genuine symmetry in any sense, formal existence and uniqueness proved sufficient to explain the magnetic flux invariant for charged particles, and more generally adiabatic invariants in any nearly periodic system.

A simple consequence of the existence of a formal hidden symmetry and corresponding adiabatic invariant for a nearly periodic Hamiltonian system is existence of a non-trivial class of phase space diffeomorphisms with hidden symmetries and adiabatic invariants. At a minimum, this class contains the time-*t* flow maps for nearly periodic Hamiltonian systems, for any value of *t*. The work presented in this article emerged from a desire to better understand this interesting class of maps. Following Kruskal’s lead, our goal was to first define these maps axiomatically and then transcribe Kruskal’s arguments to the extent possible in order to prove they have the right properties. Section 4 represents the successful culmination of this effort.

Our theory of discrete-time nearly periodic systems, or nearly periodic maps for brevity, largely parallels Kruskal’s original theory in continuous time. But there is one key technical challenge that appears in discrete time and not in continuous time, which can be described as follows. Ultimately, perturbation theory for dynamical systems involves integration along unperturbed trajectories. For a continuous-time nearly periodic system, such integrations manifest themselves in the form of partial differential equations of the general type

$$\begin{aligned} \omega (x)\,\partial _\theta f(x,\theta ) = S(x,\theta ),\quad x\in {\mathbb {R}}^n,\quad \theta \in {\mathbb {R}}\text { mod }2\pi \end{aligned}$$

where the source function $S(x,\theta )$ and the (assumed nowhere vanishing) angular frequency $\omega (x)$ are known and $f(x,\theta )$ is the dependent variable. Provided the $\theta$-average of *S* vanishes, this equation yields to solution by the method of Fourier series. No resonances appear. For nearly periodic maps, integrating along unperturbed orbits instead leads to functional equations of the form

$$\begin{aligned} f(x,\theta +\theta _0) - f(x,\theta ) = S(x,\theta ),\quad x\in {\mathbb {R}}^n,\quad \theta \in {\mathbb {R}}\text { mod }2\pi \end{aligned}$$

where $\theta _0$ is a constant parameter characterizing the map’s limiting rotation angle. Fourier analysis in $\theta$ reveals that solving for the $n^{\text {th}}$ Fourier coefficient $f_n$ requires division by $1-\exp (i\,n\,\theta )$. This step becomes problematic whenever $\theta = 2\,\pi \,q$, where *q* is a rational number. Of course, this issue may be avoided formally by choosing the limiting rotation angle to be an irrational fraction of $2\pi$. But the possibility of resonance remains in the theory, leading to a dichotomy between resonant and non-resonant nearly periodic maps. Most of Kruskal’s arguments transcribe nicely to discrete time in the non-resonant case only.

In light of our recent results from Burby and Hirvijoki (2021), existence of nearly periodic maps presented interested possibilities for important applications of our theory. For example, we showed that every Hamiltonian system on an exact symplectic manifold embeds as a slow manifold for a larger nearly periodic system with a simple Hamiltonian structure. Due to the close relationship between flow maps for nearly periodic systems and nearly periodic maps, this suggests that nearly periodic maps might be useful for simulating a very broad class of Hamiltonian systems without the need for first identifying canonical variables. Section 4.2 shows that this hunch is actually correct. Given a possibly non-canonical Hamiltonian system, we first embed the dynamics as a slow manifold in a nearly periodic Hamiltonian system using the construction from Burby and Hirvijoki (2021). Then, we construct a nearly periodic map that preserves the Hamiltonian structure of the larger system and approximately integrates the nearly periodic flow. By restricting such an integrator to initial conditions that lie on the zero level set of the map’s adiabatic invariant (guaranteed by the general theory of nearly periodic maps), we obtain an effective structure-preserving integrator for the original Hamiltonian dynamics. No canonical variables required.

## Kruskal’s Theory of Nearly Periodic Systems

In 1962, Kruskal presented an asymptotic theory (Kruskal 1962) of averaging for dynamical systems whose trajectories are all periodic to leading order. Nowadays, Kruskal’s method is termed one-phase averaging (Lochak 1993), which suggests a contrast with the multi-phase averaging methods underlying, e.g., KAM theory. Since this theory provides a model for the results in this article, we review its main ingredients here. In this section only, and merely for simplicity’s sake, we make the restriction $\Gamma ={\mathbb {R}}$.

### Definition 2

A **nearly periodic system** on a manifold *M* is a smooth $\gamma$-dependent vector field $X_\gamma$ on *M* such that $X_0 = \omega _0\,R_0$, where

- $\omega _0:M\rightarrow {\mathbb {R}}$ is strictly positive
- $R_0$ is the infinitesimal generator for a circle action $\Phi _\theta :M\rightarrow M$, $\theta \in U(1)$.
- $${\mathcal {L}}_{R_0}\omega _0 = 0$$.

The vector field $R_0$ is called the **limiting roto-rate**, and $\omega _0$ is the **limiting angular frequency**.

### Remark 1

In addition to requiring that $\omega _0$ is sign-definite, Kruskal assumed that $R_0$ is nowhere vanishing. However, this assumption is not essential for one-phase averaging to work. It is enough to require that $\omega _0$ vanishes nowhere. This is an important restriction to lift since many interesting circle actions have fixed points.

Kruskal’s theory applies to both Hamiltonian and non-Hamiltonian systems. In the Hamiltonian setting, it leads to stronger conclusions. A general class of Hamiltonian systems for which the theory works nicely may be defined as follows.

### Definition 3

Let $(M,\Omega _\gamma )$ be a manifold equipped with a smooth $\gamma$-dependent presymplectic form $\Omega _\gamma$. Assume there is a smooth $\gamma$-dependent 1-form $\vartheta _\gamma$ such that $\Omega _\gamma = - {\textbf{d}}\vartheta _\gamma$. A **nearly periodic Hamiltonian system** on $(M,\Omega _\gamma )$ is a nearly periodic system $X_\gamma$ on *M* such that $\iota _{{X}_\gamma }\Omega _\gamma = {\textbf{d}}H_\gamma$, for some smooth $\gamma$-dependent function $H_\gamma :M\rightarrow {\mathbb {R}}$.

Kruskal showed that all nearly periodic systems admit an approximate *U*(1)-symmetry that is determined to leading order by the unperturbed periodic dynamics. He named the generator of this approximate symmetry the *roto-rate*. In the Hamiltonian setting, he showed that both the dynamics and the Hamiltonian structure are *U*(1)-invariant to all orders in $\gamma$.

### Definition 4

A **roto-rate** for a nearly periodic system $X_\gamma$ on a manifold *M* is a formal power series $R_\gamma = R_0 + \gamma \,R_1 + \gamma ^2\,R_2 + \dots$ with vector field coefficients such that

- $R_0$ is equal to the limiting roto-rate
- $$\exp (2\pi {\mathcal {L}}_{R_\gamma }) = 1$$
- $$[X_\gamma ,R_\gamma ] = 0$$,

where the second and third conditions are understood in the sense of formal power series.

### Proposition 1

(Kruskal (1962)) Every nearly periodic system admits a unique roto-rate $R_\gamma$. The roto-rate for a nearly periodic Hamiltonian system on an exact presymplectic manifold $(M,\Omega _\gamma )$ satisfies ${\mathcal {L}}_{R_\gamma }\Omega _\gamma = 0$ in the sense of formal power series.

### Corollary 1

The roto-rate $R_\gamma$ for a nearly periodic Hamiltonian system $X_\gamma$ on an exact presymplectic manifold $(M,\Omega _\gamma )$ with Hamiltonian $H_\gamma$ satisfies ${\mathcal {L}}_{R_\gamma }H_\gamma = 0$.

### Proof

Since $[R_\gamma ,X_\gamma ] = {\mathcal {L}}_{R_\gamma }X_\gamma = 0$ and ${\mathcal {L}}_{R_\gamma }\Omega _\gamma = 0$, we may apply the Lie derivative ${\mathcal {L}}_{R_\gamma }$ to Hamilton’s equation $\iota _{X_\gamma }\Omega _\gamma = {\textbf{d}}H_\gamma$ to obtain

$$\begin{aligned} {\mathcal {L}}_{R_\gamma }({\textbf{d}}H_\gamma ) = {\mathcal {L}}_{R_\gamma }(\iota _{X_\gamma }\Omega _\gamma ) = \iota _{{\mathcal {L}}_{R_\gamma } X_\gamma }\Omega _\gamma + \iota _{X_\gamma }({\mathcal {L}}_{R_\gamma }\Omega _\gamma ) = 0. \end{aligned}$$

Thus, ${\mathcal {L}}_{R_\gamma }H_\gamma$ is a constant function. By averaging over the *U*(1)-action we conclude that the constant must be zero. $\square$

To prove Proposition 1, Kruskal used a pair of technical results, each of which is interesting in its own right. The first establishes the existence of a non-unique normalizing transformation that asymptotically deforms the *U*(1)-action generated by $R_\gamma$ into the simpler *U*(1)-action generated by $R_0$. The second is a subtle bootstrapping argument that upgrades leading-order *U*(1)-invariance to all-orders *U*(1)-invariance for integral invariants. We state these results here for future reference.

### Definition 5

Let $G_\gamma =\gamma \,G_1+ \gamma ^2\,G_2 + \dots$ be an $O(\gamma )$ (no constant term) formal power series whose coefficients are vector fields on a manifold *M*. The **Lie transform** with **generator** $G_\gamma$ is the formal power series $\exp ({\mathcal {L}}_{G_\gamma })$ whose coefficients are differential operators on the tensor algebra over *M*.

### Definition 6

A **normalizing transformation** for a nearly periodic system $X_\gamma$ with roto-rate $R_\gamma$ is a Lie transform $\exp ({\mathcal {L}}_{G_\gamma })$ with generator $G_\gamma$ such that $R_\gamma = \exp ({\mathcal {L}}_{G_\gamma })R_0$.

### Proposition 2

(Kruskal) Each nearly periodic system admits a normalizing transformation.

### Proposition 3

Let $\alpha _\gamma$ be a smooth $\gamma$-dependent differential form on a manifold *M*. Suppose $\alpha _\gamma$ is an absolute integral invariant for a $C^\infty$ nearly periodic system $X_\gamma$ on *M*. If ${\mathcal {L}}_{R_0}\alpha _0 = 0$ then ${\mathcal {L}}_{R_\gamma }\alpha _\gamma = 0$, where $R_\gamma$ is the roto-rate for $X_\gamma$.

### Proof

Integral invariance means ${\mathcal {L}}_{X_\gamma }\alpha _\gamma = 0$ for each $\gamma \in \Gamma$. By Applying ${\mathcal {L}}_{R_\gamma }$ to this relationship, and using $[R_\gamma ,X_\gamma ]=0$, we obtain ${\mathcal {L}}_{X_\gamma }{\mathcal {L}}_{R_\gamma }\alpha _\gamma = 0$. Now let $G_\gamma$ be the generator of a normalizing transformation for $X_\gamma$, and set ${\overline{X}}_\gamma = \exp (-{\mathcal {L}}_{G_\gamma })X_\gamma$, ${\overline{\alpha }}_\gamma = \exp (-{\mathcal {L}}_{G_\gamma })\alpha _\gamma$. We have ${\mathcal {L}}_{{\overline{X}}_\gamma }{\mathcal {L}}_{R_0}{\overline{\alpha }}_\gamma =0$. Since ${\mathcal {L}}_{R_0}{\overline{\alpha }}_\gamma = O(\gamma )$, the first-order consequence of the previous formula is ${\mathcal {L}}_{\omega _0\,R_0}{\mathcal {L}}_{R_0}{\overline{\alpha }}_1=0$, which can only be satisfied if ${\mathcal {L}}_{R_0}{\overline{\alpha }}_1$ is $R_0$-invariant. But since the *U*(1)-average of ${\mathcal {L}}_{R_0}{\overline{\alpha }}_1$ vanishes, we conclude ${\mathcal {L}}_{R_0}{\overline{\alpha }}_1=0$. Repeating this argument gives ${\mathcal {L}}_{R_0}{\overline{\alpha }}_k=0$ for $k > 1$ as well. In other words ${\mathcal {L}}_{R_0}{\overline{\alpha }}_\gamma =0$ to all orders in $\gamma$, which is equivalent to the theorem’s claim. $\square$

According to Noether’s celebrated theorem, a Hamiltonian system that admits a continuous family of symmetries also admits a corresponding conserved quantity. Therefore, one might expect that a Hamiltonian system with approximate symmetry must also have an approximate conservation law. Kruskal showed that this is indeed the case for nearly periodic Hamiltonian systems, as the following generalization of his argument shows.

### Proposition 4

Let $X_\gamma$ be a nearly periodic Hamiltonian system on the exact presymplectic manifold $(M,\Omega _\gamma )$. Let $R_\gamma$ be the associated roto-rate. There is a formal power series $\theta _\gamma = \theta _0 + \gamma \,\theta _1 + \dots$ with coefficients in $\Omega ^1(M)$ such that $\Omega _\gamma = -{\textbf{d}}\theta _\gamma$ and ${\mathcal {L}}_{R_\gamma }\theta _\gamma = 0$. Moreover, the formal power series $\mu _\gamma = \iota _{R_\gamma }\theta _\gamma$ is a constant of motion for $X_\gamma$ to all orders in perturbation theory. In other words,

$$\begin{aligned} {\mathcal {L}}_{X_\gamma }\mu _\gamma = 0, \end{aligned}$$

in the sense of formal power series.

### Proof

To construct the *U*(1)-invariant primitive $\theta _\gamma$, we select an arbitrary primitive $\vartheta _\gamma$ for $\Omega _\gamma$ and set

$$\begin{aligned} \theta _\gamma = \frac{1}{2\pi }\int _0^{2\pi }\exp (\theta {\mathcal {L}}_{R_\gamma })\vartheta _\gamma \,d\theta . \end{aligned}$$

This formal power series satisfies ${\mathcal {L}}_{R_\gamma }\theta _\gamma =0$ because

$$\begin{aligned} {\mathcal {L}}_{R_\gamma }\theta _\gamma = \frac{1}{2\pi }\int _0^{2\pi }\frac{d}{d\theta }\exp (\theta {\mathcal {L}}_{R_\gamma })\vartheta _\gamma \,d\theta = 0. \end{aligned}$$

Moreover, since ${\mathcal {L}}_{R_\gamma }\Omega _\gamma = 0$ by Kruskal’s Proposition 1, we have

$$\begin{aligned} -{\textbf{d}}\theta _\gamma = \frac{1}{2\pi }\int _0^{2\pi }\exp (\theta {\mathcal {L}}_{R_\gamma })\Omega _\gamma \,d\theta = \frac{1}{2\pi }\int _0^{2\pi }\Omega _\gamma \,d\theta = \Omega _\gamma , \end{aligned}$$

whence $\theta _\gamma$ is a primitive for $\Omega _\gamma$.

To establish all-orders time independence of $\mu _\gamma = \iota _{R_\gamma }\theta _\gamma$, we apply Cartan’s formula and Corollary 1 according to

$$\begin{aligned} {\mathcal {L}}_{X_\gamma }\mu _\gamma = \iota _{X_\gamma }{\textbf{d}}\iota _{R_\gamma }\theta _\gamma = - \iota _{R_\gamma }\iota _{X_\gamma }\Omega _\gamma = - {\mathcal {L}}_{R_\gamma }H_\gamma = 0. \end{aligned}$$

$$\square $$

### Definition 7

The formal constant of motion $\mu _\gamma$ provided by Proposition 4 is the **adiabatic invariant** associated with a nearly periodic Hamiltonian system.

## Nearly Periodic Maps

Nearly periodic maps are natural discrete-time analogs of nearly periodic systems. The following provides a precise definition.

### Definition 8

(nearly periodic map) Let $\Gamma$ be a vector space. A **nearly periodic map** on a manifold *M* with parameter space $\Gamma$ is a smooth mapping $F:M\times \Gamma \rightarrow M$ such that $F_\gamma :M\rightarrow M:m\mapsto F(m,\gamma )$ has the following properties:

- $F_\gamma$ is a diffeomorphism for each $\gamma \in \Gamma$.
- There exists a *U*(1)-action $\Phi _\theta :M\rightarrow M$ and a constant $\theta _0\in U(1)$ such that $F_0 = \Phi _{\theta _0}$.

We say *F* is **resonant** if $\theta _0$ is a rational multiple of $2\pi$, otherwise *F* is **non-resonant**. The infinitesimal generator of $\Phi _\theta$, $R_0$, is the **limiting roto-rate**.

Let *X* be a vector field on a manifold *M* with time-*t* flow map ${\mathcal {F}}_t$. A *U*(1)-action $\Phi _\theta$ is a symmetry for *X* if ${\mathcal {F}}_t\circ \Phi _\theta = \Phi _\theta \circ {\mathcal {F}}_t$, for each $t\in {\mathbb {R}}$ and $\theta \in U(1)$. Differentiating this condition with respect to $\theta$ at the identity implies ${\mathcal {F}}_t^*R = R$, where *R* denotes the infinitesimal generator for the *U*(1)-action. Conversely, if *R* is any vector field with $2\pi$-periodic orbits and ${\mathcal {F}}_t^*R = R$ for each $t\in {\mathbb {R}}$ then the *R*-flow defines a *U*(1)-action that is a symmetry for *X*. Since we would like to think of nearly periodic maps as playing the part of a nearly periodic system’s flow map, the latter characterization of symmetry allows us to naturally extend Kruskal’s notion of roto-rate to our discrete-time setting.

### Definition 9

A **roto-rate** for a nearly periodic map *F* is a formal power series $R_\gamma = R_0 + R_1[\gamma ] + R_2[\gamma ,\gamma ]+\dots$ whose coefficients are homogeneous polynomial maps from $\Gamma$ into vector fields on *M* such that

- $R_0$ is the limiting roto-rate.
- $F_\gamma ^*R_\gamma = R_\gamma$ in the sense of formal power series.
- $\exp (2\pi {\mathcal {L}}_{R_\gamma })=1$ in the sense of formal power series.

### Definition 10

Let $G_\gamma =G_1[\gamma ]+ G_2[\gamma ,\gamma ] + \dots$ be an $O(\gamma )$ (no constant term) formal power series whose coefficients are homogeneous polynomial maps from $\Gamma$ into vector fields on *M*. The **Lie transform** with **generator** $G_\gamma$ is the formal power series $\exp ({\mathcal {L}}_{G_\gamma })$ whose coefficients are homogeneous polynomial maps from $\Gamma$ into differential operators on the tensor algebra over *M*.

### Remark 2

Since the parameter $\gamma$ in the previous two definitions is vector valued, the formal power series $R_\gamma$ and $G_\gamma$ may be usefully interpreted as multivariate formal power series.

### Definition 11

A **normalizing transformation** for a nearly periodic map *F* with roto-rate $R_\gamma$ is a Lie transform $\exp ({\mathcal {L}}_{G_\gamma })$ with generator $G_\gamma$ such that $R_\gamma = \exp ({\mathcal {L}}_{G_\gamma })R_0$.

Our first and most fundamental result concerning nearly periodic maps establishes the existence and uniqueness of the roto-rate in the non-resonant case. Like the corresponding result due to Kruskal in continuous time, this result holds to all orders in perturbation theory.

### Theorem 1

(Existence and uniqueness of the roto-rate) Each non-resonant nearly periodic map admits a unique roto-rate.

### Proof

First we will show that there exists a Lie transform with generator $G_\gamma$ such that $R_\gamma \equiv \exp ({\mathcal {L}}_{G_\gamma })R_0$ is a roto-rate.

To that end, we introduce a convenient way of representing $\gamma$-dependent pullback operators at the level of formal power series. Let $\psi _\gamma$ be a smooth $\gamma$-dependent diffeomorphism on *M*. By the Lie derivative formula, there is a unique $\gamma$-dependent $\Gamma ^*$-valued vector field $W_\gamma$ such that

1 $$\begin{aligned} \left. \frac{d}{ds}\right| _{s=0}\psi _{\gamma +s\,\delta \gamma }^* = \psi _\gamma ^*{\mathcal {L}}_{\langle W_\gamma ,\delta \gamma \rangle }, \end{aligned}$$

for each $\gamma ,\delta \gamma \in \Gamma$. Here $\langle \cdot ,\cdot \rangle$ denotes the natural pairing between $\Gamma$ and its dual space $\Gamma ^*$. The object $W_\gamma$ both determines and is determined by the pullback operator $\psi _\gamma ^*$ at the level of formal power series. This follows from recursive application of the identity

2 $$\begin{aligned} \psi _{s\gamma }^* = \psi _0^* + \int _0^s\psi _{s_1\gamma }^*{\mathcal {L}}_{\langle W_{s_1\,\gamma },\gamma \rangle }\,ds_1, \end{aligned}$$

which may be understood as a consequence of (1) and the fundamental theorem of calculus. This can be viewed as Picard iteration of (1) or fixed point iteration of (2). The first step in the recursion is to substitute (2) with $s=s_1$ into (2) with $s=1$, resulting in

$$\begin{aligned} \psi _{\gamma }^*&= \psi _0 + \int _0^1\left( \psi _0^* + \int _0^{s_1}\psi _{s_2\gamma }^*{\mathcal {L}}_{\langle W_{s_2\,\gamma },\gamma \rangle }\,ds_2 \right) {\mathcal {L}}_{\langle W_{s_1\,\gamma },\gamma \rangle }\,ds_1\\&= \psi _0 + \int _0^1\psi _0^* {\mathcal {L}}_{\langle W_{s_1\,\gamma },\gamma \rangle }\,ds_1 + \int _0^1\int _0^{s_1}\psi _{s_2\gamma }^*{\mathcal {L}}_{\langle W_{s_2\,\gamma },\gamma \rangle } {\mathcal {L}}_{\langle W_{s_1\,\gamma },\gamma \rangle }\,ds_2\,ds_1. \end{aligned}$$

The second step involves substituting (2) with $s=s_2$ into the preceding expression, thereby producing a triple integral. Continuing in this manner, it is straightforward to derive the following time-ordered exponential formulas for both the pullback $\psi _\gamma ^*$ and pushforward operator $\psi _{\gamma *}$,

3 $$\begin{aligned} \psi _\gamma ^*&= \psi _0^*\left[ 1 + \int _0^1 {\mathcal {L}}_{\langle W_{s_1\,\gamma },\gamma \rangle }\,ds_1 + \int _0^1\int _0^{s_1}{\mathcal {L}}_{\langle W_{s_2\,\gamma },\gamma \rangle }{\mathcal {L}}_{\langle W_{s_1\,\gamma },\gamma \rangle }\,ds_2\,ds_1 +\dots \right] \end{aligned}$$

4 $$\begin{aligned} \psi _{\gamma *}&= \left[ 1 - \int _0^1 {\mathcal {L}}_{\langle W_{s_1\,\gamma },\gamma \rangle }\,ds_1 + \int _0^1\int _0^{s_1}{\mathcal {L}}_{\langle W_{s_1\,\gamma },\gamma \rangle }{\mathcal {L}}_{\langle W_{s_2\,\gamma },\gamma \rangle }\,ds_2\,ds_1+\dots \right] \,\psi _{0\,*}. \end{aligned}$$

Upon introducing the formal power series expansion $W_\gamma = W_0 + W_1[\gamma ] + W_2[\gamma ,\gamma ] + \dots$, the integrals in these formulas can be carried out, leading to the somewhat more explicit formulas

5 $$\begin{aligned} \psi _\gamma ^*&=\psi _0^*\left[ 1 + {\mathcal {L}}_{\langle W_0,\gamma \rangle } + \frac{1}{2}{\mathcal {L}}_{\langle W_1[\gamma ],\gamma \rangle } + \frac{1}{2}{\mathcal {L}}^2_{\langle W_0,\gamma \rangle }+O(\gamma ^3)\right] \end{aligned}$$

6 $$\begin{aligned} \psi _{\gamma *}&= \left[ 1 - {\mathcal {L}}_{\langle W_0,\gamma \rangle } - \frac{1}{2}{\mathcal {L}}_{\langle W_1[\gamma ],\gamma \rangle } + \frac{1}{2}{\mathcal {L}}^2_{\langle W_0,\gamma \rangle }+O(\gamma ^3)\right] \,\psi _{0\,*}. \end{aligned}$$

The preceding discussion applies in particular to $\psi _\gamma ^* = F_\gamma ^*$. In this case, we will use the symbol $V_\gamma$ for $W_\gamma$. The discussion also applies to the formal pullback operator $\psi _\gamma ^* = \phi _\gamma ^*$, where

$$\begin{aligned} \phi _\gamma ^* = \exp ({\mathcal {L}}_{G_\gamma }), \end{aligned}$$

as well as its inverse $\phi _{\gamma *} = (\phi _\gamma ^*)^{-1}$. In this case we will use $\xi _\gamma$ in place of $W_\gamma$. Thus, we have the defining identities

7 $$\begin{aligned} \frac{d}{ds}\bigg |_{s=0}F_{\gamma +s\,\delta \gamma }^*&= F_\gamma ^*{\mathcal {L}}_{\langle V_\gamma ,\delta \gamma \rangle } \end{aligned}$$

8 $$\begin{aligned} \frac{d}{ds}\bigg |_{s=0}\phi _{\gamma +s\,\delta \gamma }^*&= \phi _\gamma ^*{\mathcal {L}}_{\langle \xi _\gamma ,\delta \gamma \rangle }. \end{aligned}$$

We will now establish existence of the Lie transform with generator $G_\gamma$ by constructing an appropriate $\xi _\gamma$. The Lie transform itself can then be constructed using the formulas (3) and (4). Define $R_\gamma = \exp ({\mathcal {L}}_{G_\gamma })R_0 = \phi _\gamma ^*R_0$, where $G_\gamma$, or equivalently $\xi _\gamma$, is yet to be determined. This $R_\gamma$ satisfies $\exp (2\pi {\mathcal {L}}_{R_\gamma })=1$ and $R_{\gamma =0}=R_0$ automatically. We will determine the formal power series $\xi _\gamma = \xi _0 + \xi _1[\gamma ] +\xi _2[\gamma ,\gamma ]+\dots$ by requiring $F_\gamma ^*R_\gamma = R_\gamma$. If this can be done, then $R_\gamma$ will be a roto-rate.

The equation we would like to solve is equivalent to

9 $$\begin{aligned} (\phi _\gamma ^{-1})^*F_\gamma ^*\phi _\gamma ^*R_0 = R_0, \end{aligned}$$

where $(\phi _\gamma ^{-1})^* = (\phi _\gamma ^*)^{-1}$. Formally, this is just the statement that the “diffeomorphism” ${\overline{F}}_\gamma = \phi _\gamma \circ F_\gamma \circ \phi _\gamma ^{-1}$ preserves the limiting roto-rate $R_0$. Instead of solving (9) directly, we will demand that its $\gamma$-derivative vanishes. This derivative condition is

10 $$\begin{aligned} 0 = \frac{d}{ds}\bigg |_{s=0}{\overline{F}}_{\gamma +s\,\delta \gamma }^*R_0 = {\overline{F}}_\gamma ^*{\mathcal {L}}_{\langle {\overline{V}}_\gamma ,\delta \gamma \rangle }R_0, \end{aligned}$$

where ${\overline{V}}_\gamma$ is readily shown to be given by

$$\begin{aligned} {\overline{V}}_\gamma = \xi _\gamma - {\overline{F}}_{\gamma \,*}\xi _\gamma + \phi _{\gamma \,*}V_\gamma . \end{aligned}$$

Note that requiring the $\gamma$-derivative of (9) to vanish implies (9) itself since the latter is clearly satisfied when $\gamma =0$. Also note that since ${\overline{F}}_\gamma ^*$ is formally invertible, the derivative condition is equivalent to

11 $$\begin{aligned} {\mathcal {L}}_{R_0}{\overline{V}}_\gamma = 0. \end{aligned}$$

To solve (11), we will expand the equation in powers of $\gamma$ and then argue inductively that each equation in the resulting sequence can be solved. At $O(\gamma ^0)$ we have

$$\begin{aligned} {\mathcal {L}}_{R_0}(\xi _0 - \Phi _{\theta _0\,*}\xi _0 + V_0) = 0. \end{aligned}$$

Denoting the limiting *U*(1)-average operation as $\langle {\varvec{T}} \rangle = (2\pi )^{-1}\int _0^{2\pi }\Phi _\theta ^*{\varvec{T}}\,d\theta$, where ${\varvec{T}}$ is any tensor field on *M*, and ${\varvec{T}}^{\text {osc}} = {\varvec{T}} - \langle {\varvec{T}}\rangle$, this equation is equivalent to

$$\begin{aligned} A_{\theta _0}\xi _0^{\text {osc}} = -V_0^{\text {osc}}, \end{aligned}$$

where we have introduced the **homological operator**

$$\begin{aligned} A_{\theta _0} = 1 - \Phi _{\theta _0\,*}. \end{aligned}$$

Since $F_\gamma$ is assumed to be non-resonant, the homological operator, regarded as a linear automorphism of the oscillating subspace of vector fields, is invertible. We may therefore solve the $O(\gamma ^0)$ equation by setting

$$\begin{aligned} \xi _0&= -A_{\theta _0}^{-1}V_0^{\text {osc}}. \end{aligned}$$

At $O(\gamma ^n)$, (11) leads to

$$\begin{aligned} A_{\theta _0}\xi _n[\gamma ,\dots ,\gamma ]^{\text {osc}} = S_n[\gamma ,\dots ,\gamma ]^{\text {osc}}, \end{aligned}$$

where $S_n[\gamma ,\dots ,\gamma ]$ involves coefficients of $V_\gamma = V_0 + V_1[\gamma ] + V_2[\gamma ,\gamma ]$ and $\xi _\gamma = \xi _0 + \xi _1[\gamma ] + \xi _2[\gamma ,\gamma ] + \dots$ with polynomial degree (for $\xi$) at most $n-1$. Assuming the $\xi _k$ with $k < n$ have already been determined by solving the $O(\gamma ^k)$ components of (11), we may therefore solve the $O(\gamma ^n)$ equation by setting

$$\begin{aligned} \xi _n[\gamma ,\dots ,\gamma ] = A_{\theta _0}^{-1}S_n[\gamma ,\dots ,\gamma ]^{\text {osc}}. \end{aligned}$$

Since we have already established that the $O(\gamma ^0)$ equation has a solution, we now conclude by induction that (11) may be solved for $\xi _\gamma$ to all orders in $\gamma$. It follows that a roto-rate exists.

Next we prove uniqueness of the $R_\gamma$ just constructed. Suppose $R_\gamma ^\prime$ is a possibly distinct roto-rate, and consider the commutator $C_\gamma = [R_\gamma ,R_\gamma ^\prime ]$. Immediate properties of $C_\gamma$ include $C_0 = 0$ and $F_\gamma ^*C_\gamma = C_\gamma$. By construction of $R_\gamma$, we have $R_\gamma = \exp ({\mathcal {L}}_{G_\gamma })R_0$, which implies ${\overline{C}}_\gamma = \exp (-{\mathcal {L}}_{G_\gamma })C_\gamma = [R_0,{\overline{R}}_\gamma ^\prime ]$, where ${\overline{R}}_\gamma ^\prime = \exp (-{\mathcal {L}}_{G_\gamma })R_\gamma ^\prime$. Thus, the mean $\langle {\overline{C}}_\gamma \rangle =0$ vanishes to all orders in $\gamma$. Since $F_\gamma ^*C_\gamma = C_\gamma$, we also have ${\overline{F}}_\gamma ^* {\overline{C}}_\gamma = {\overline{C}}_\gamma$, where ${\overline{F}}_\gamma ^*\equiv \exp (-{\mathcal {L}}_{G_\gamma })F_\gamma ^*\exp ({\mathcal {L}}_{G_\gamma })$. The first-order consequence of the last condition is $\Phi _{\theta _0}^*{\overline{C}}_1[\gamma ] = {\overline{C}}_1[\gamma ]$, which implies ${\mathcal {L}}_{R_0}{\overline{C}}_1[\gamma ] = 0$ by non-resonance. But since the mean of ${\overline{C}}_1[\gamma ]$ vanishes, we must have ${\overline{C}}_1[\gamma ]=0$ for all $\gamma \in \Gamma$. The same argument applied repeatedly implies ${\overline{C}}_n=0$ for all $n\ge 1$. In other words, $R_\gamma$ and $R_\gamma ^\prime$ commute. To finish the argument for uniqueness, we now use commutativity of $R_\gamma$ and $R_\gamma ^\prime$ to find $1=\exp (2\pi {\mathcal {L}}_{R_\gamma }) = \exp (2\pi {\mathcal {L}}_{R_\gamma ^\prime } + 2\pi {\mathcal {L}}_{R_\gamma - R_\gamma ^\prime }) = \exp (2\pi {\mathcal {L}}_{R_\gamma - R_\gamma ^\prime }),$ which can only be satisfied if $R_\gamma - R_\gamma ^\prime = 0$, since $R_0 - R_0^\prime =0$.

$$\square $$

### Theorem 2

(Existence of normalizing transformations) Each non-resonant nearly periodic map admits a normalizing transformation.

### Proof

This follows immediately from the proof of Theorem 1. $\square$

### Nearly Periodic Maps With Hamiltonian Structure

As in the continuous-time theory, existence of the roto-rate leads to additional insights for nearly periodic maps that are Hamiltonian in an appropriate sense. In this subsection, we will establish the basic properties of nearly periodic maps with Hamiltonian structure. We start by defining what we mean by Hamiltonian structure.

#### Definition 12

(Presymplectic nearly periodic map) A **Presymplectic nearly periodic map** on a $\gamma$-dependent presymplectic manifold $(M,\Omega _\gamma )$ is a nearly periodic map *F* such that $F_\gamma ^*\Omega _\gamma = \Omega _\gamma$ for each $\gamma \in \Gamma$.

#### Definition 13

(Hamiltonian nearly periodic map) A **Hamiltonian nearly periodic map** on a $\gamma$-dependent presymplectic manifold $(M,\Omega _\gamma )$ is a nearly periodic map *F* such that there is a smooth $(t,\gamma )$-dependent vector field $Y_{t,\gamma }$ with the following properties:

- $$t\in {\mathbb {R}}$$.
- $\iota _{Y_{t,\gamma }}\Omega _\gamma = {\textbf{d}}H_{t,\gamma }$, for some smooth $(t,\gamma )$-dependent function $H_{t,\gamma }$.
- For each $\gamma \in \Gamma$, $F_\gamma$ is the $t=1$ flow of $Y_{t,\gamma }$.

#### Lemma 1

Each Hamiltonian nearly periodic map is a presymplectic nearly periodic map.

#### Theorem 3

(Roto-rate is presymplectic) If *F* is a non-resonant presymplectic nearly periodic map on a $\gamma$-dependent presymplectic manifold $(M,\Omega _\gamma )$ with roto-rate $R_\gamma$ then ${\mathcal {L}}_{R_\gamma }\Omega _\gamma = 0$.

#### Proof

First note that presymplecticity of *F* with $\gamma =0$ implies $F_0^*\Omega _0 =\Phi _{\theta _0}^*\Omega _0= \Omega _0$. Upon introducing the 2-form-valued Fourier coefficients,

12 $$\begin{aligned} \Omega _0^k = \frac{1}{2\pi }\int _0^{2\pi }e^{-ik\theta }\Phi _\theta ^*\Omega _0\,d\theta ,\quad k\in {\mathbb {Z}}, \end{aligned}$$

the last identity may be rewritten as the sequence of identities $e^{ik\theta _0}\Omega _0^k = \Omega _0^k$, $k\in {\mathbb {Z}}$. But by non-resonance of *F*, $1-e^{ik\theta _0}$ is nonvanishing for each *k*. We conclude that $\Omega _0^k=0$ for nonzero *k*, or, equivalently, ${\mathcal {L}}_{R_0}\Omega _0 = 0$.

Presymplecticity of *F* for nonzero $\gamma$ implies $F_\gamma ^*\Omega _\gamma = \Omega _\gamma$ for each $\gamma \in \Gamma$. Applying the Lie derivative ${\mathcal {L}}_{R_\gamma }$ to this identity and using $F_\gamma ^*R_\gamma = R_\gamma$ implies $F_\gamma ^*({\mathcal {L}}_{R_\gamma }\Omega _\gamma ) =({\mathcal {L}}_{R_\gamma }\Omega _\gamma )$. In other words, $\alpha _\gamma = {\mathcal {L}}_{R_\gamma }\Omega _\gamma$ is (formally) an absolute integral invariant for $F_\gamma$. By the argument from the previous paragraph, we see immediately that $\alpha _0 = 0$. To finish the proof, we will use integral invariance together with existence of a normalizing transformation to find that $\alpha _\gamma =0$ to all orders in $\gamma$. This argument will parallel the proof of Proposition 3.

Let $G_\gamma$ be the generator of a normalizing transformation for *F* given by Theorem 2. Set ${\overline{\alpha }}_\gamma = \exp (-{\mathcal {L}}_{G_\gamma })\alpha _\gamma = {\overline{\alpha }}_0 + {\overline{\alpha }}_1[\gamma ] +{\overline{\alpha }}_2[\gamma ,\gamma ] +\dots$. Note that $\alpha _\gamma = 0$ if and only if ${\overline{\alpha }}_\gamma =0$. Since $\alpha _\gamma$ is an integral invariant for $F_\gamma$, ${\overline{\alpha }}_\gamma$ must satisfy

13 $$\begin{aligned} \exp (-{\mathcal {L}}_{G_\gamma })F_\gamma ^*\exp ({\mathcal {L}}_{G_\gamma }){\overline{\alpha }}_\gamma = {\overline{\alpha }}_\gamma .\end{aligned}$$

Because ${\overline{\alpha }}_0 = \alpha _0 = 0$, the first-order consequence of (13) is $F_0^*{\overline{\alpha }}_1[\gamma ] = \Phi _{\theta _0}^*{\overline{\alpha }}_1[\gamma ] ={\overline{\alpha }}_1[\gamma ]$. By our earlier argument, we must then have $({\overline{\alpha }}_1[\gamma ])^k=0$ for $k\ne 0$. But using $\exp (-{\mathcal {L}}_{G_\gamma })R_\gamma = R_0$, we also find ${\overline{\alpha }}_\gamma = {\mathcal {L}}_{R_0}{\overline{\Omega }}_\gamma$, where ${\overline{\Omega }}_\gamma = \exp (-{\mathcal {L}}_{G_\gamma })\Omega _\gamma$. The latter implies ${\overline{\alpha }}_\gamma ^0 = 0$, and $({\overline{\alpha }}_1[\gamma ])^0=0$ in particular. Thus, ${\overline{\alpha }}_1[\gamma ]=0$ for all $\gamma \in \Gamma$. We may now repeat this argument for ${\overline{\alpha }}_2[\gamma ,\gamma ]$, ${\overline{\alpha }}_3[\gamma ,\gamma ,\gamma ]$, etc., to obtain the desired result. $\square$

Using presymplecticity of the roto-rate, we may now use a version of Noether’s theorem to establish existence of adiabatic invariants for many interesting presymplectic nearly periodic maps.

#### Theorem 4

(Existence of an adiabatic invariant) Let *F* be a non-resonant presymplectic nearly periodic map on the exact $\gamma$-dependent presymplectic manifold $(M,\Omega _\gamma )$ with roto-rate $R_\gamma$. Assume one of the following conditions is satisfied.

1. *F* is Hamiltonian.
2. *M* is connected and the limiting roto-rate $R_0$ has at least one zero.

There exists a smooth $\gamma$-dependent 1-form $\theta _\gamma$ such that ${\mathcal {L}}_{R_\gamma }\theta _\gamma = 0$ and $-{\textbf{d}}\theta _\gamma =\Omega _\gamma$ in the sense of formal power series. Moreover the quantity

14 $$\begin{aligned} \mu _\zeta = \iota _{R_\gamma }\theta _\gamma \end{aligned}$$

satisfies $F_\gamma ^*\mu _\gamma = \mu _\gamma$ in the sense of formal power series. In other words, $\mu _\gamma$ is an adiabatic invariant for *F*.

#### Proof

By Theorem 3, a primitive $\theta _\gamma$ with the desired properties may be constructed as in the proof of Proposition 4.

To establish adiabatic invariance of $\mu _\gamma$, first we compute the exterior derivative of $\mu _\gamma$ using Cartan’s formula to obtain ${\textbf{d}}\mu _\gamma = \iota _{R_\gamma }\Omega _\gamma$. Since both $R_\gamma$ and $\Omega _\gamma$ are $F_\gamma$-invariant, it follows that ${\textbf{d}}F_\gamma ^*\mu _\gamma = {\textbf{d}}\mu _\gamma$. The difference $c_\gamma \equiv F_\gamma ^*\mu _\gamma - \mu _\gamma$ must therefore be a formal power series whose coefficients are homogeneous polynomial maps from $\Gamma$ into locally constant functions on *M*. To complete the proof, we must demonstrate that $c_\gamma = 0$ to all orders.

First suppose *F* is Hamiltonian. Then, there is a smooth $(t,\gamma )$-dependent Hamiltonian vector field $Y_{t,\gamma }$ with Hamiltonian $H_{t,\gamma }$ whose $t=1$ flow is equal to $F_\gamma$. Let ${\mathcal {F}}^\gamma _t$ denote the time-*t* flow map for $Y_{t,\gamma }$ with ${\mathcal {F}}^\gamma _0 = \text {id}_M$. By the fundamental theorem of calculus, the definition of Lie derivative, and Cartan’s formula, we therefore have

$$\begin{aligned} F_\gamma ^*\mu _\gamma&= \iota _{R_\gamma }({\mathcal {F}}^\gamma _1)^*\theta _\gamma \\&= \iota _{R_\gamma }\theta _\gamma +\iota _{R_\gamma }\int _0^1 \frac{d}{dt} ({\mathcal {F}}^\gamma _t)^*\theta _\gamma \,dt\\&= \mu _\gamma + \iota _{R_\gamma }\int _0^1 ({\mathcal {F}}^\gamma _t)^*({\mathcal {L}}_{Y_{t,\gamma }}\theta _\gamma )\,dt\\&= \mu _\gamma + \iota _{R_\gamma }\int _0^1 ({\mathcal {F}}^\gamma _t)^*(\iota _{Y_{t,\gamma }}{\textbf{d}}\theta _\gamma + {\textbf{d}}\iota _{Y_{t,\gamma }}\theta _\gamma )\,dt\\&= \mu _\gamma + \iota _{R_\gamma }\int _0^1 ({\mathcal {F}}^\gamma _t)^* {\textbf{d}}(\iota _{Y_{t,\gamma }}\theta _\gamma -H_{t,\gamma })\,dt\\&= \mu _\gamma + {\mathcal {L}}_{R_\gamma }\int _0^1 ({\mathcal {F}}^\gamma _t)^* (\iota _{Y_{t,\gamma }}\theta _\gamma -H_{t,\gamma })\,dt. \end{aligned}$$

Applying $\exp (\theta {\mathcal {L}}_{R_0})$ to this identity and averaging in $\theta$ gives the desired result.

Finally suppose that *M* is connected and that $R_0(m_0)=0$ for some $m_0\in M$. Let $G_\gamma$ be the generator of a normalizing transformation. We have

$$\begin{aligned} \exp (-{\mathcal {L}}_{G_\gamma })\mu _\gamma (m_0) = \iota _{R_0}\exp (-{\mathcal {L}}_{G_\gamma }){\theta }_\gamma (m_0)=0, \end{aligned}$$

and

$$\begin{aligned} \exp (-{\mathcal {L}}_{G_\gamma })F_\gamma ^*\mu _\gamma (m_0)&= \exp (-{\mathcal {L}}_{G_\gamma })\iota _{R_\gamma }F_\gamma ^*\theta _\gamma (m_0) = \iota _{R_0}(\exp (-{\mathcal {L}}_{G_\gamma })F_\gamma ^*\theta _\gamma )(m_0)=0. \end{aligned}$$

It follows that $c_\gamma$ is zero on the connected component of *M* containing $m_0$. But since *M* is connected, $c_\gamma$ is therefore zero everywhere, as claimed. $\square$

#### Remark 3

A simple example illustrates how existence of an adiabatic invariant can fail. Let $M =S^1\times {\mathbb {R}} \ni (\zeta ,I)$, $\Omega _\gamma = d\zeta \wedge dI = -{\textbf{d}}(I\,d\zeta )$, and $\Gamma ={\mathbb {R}}$. The mapping $F(\zeta ,I,\gamma ) = (\zeta +\theta _0,I+\gamma )$ defines a non-resonant nearly periodic map for almost all $\theta _0\in U(1)$. The roto-rate is given to all orders by $R_\gamma = \partial _\zeta$. Moreover, $F_\gamma$ is area-preserving for each $\gamma$, and hence presymplectic. The quantity $\mu _\gamma = \iota _{R_\gamma }(I\,d\zeta ) = I$ from (14) is clearly not an adiabatic invariant for *F* since $F_\gamma ^*I = I + \gamma$. Note that, in this example, the $R_0$-orbits are not contractible and that *F* is presymplectic but not Hamiltonian.

### Geometric Integration of Noncanonical Hamiltonian Systems Using Nearly Periodic Maps

Let $Q\subset E$ be a connected open subset of a finite-dimensional vector space *E* equipped with an exact symplectic form $\omega = -{\textbf{d}}\vartheta$. Consider a Hamiltonian system on $(Q,\omega =-{\textbf{d}}\vartheta )$ with Hamiltonian $H:Q\rightarrow {\mathbb {R}}$. Choose an almost complex structure ${\mathbb {J}}:TQ\rightarrow TQ$ compatible with the symplectic form $\omega = -{\textbf{d}}\vartheta$, so that $g(v,w) = \omega (v,{\mathbb {J}}w)$ defines a metric tensor on *Q*. Equip the tangent bundle $\pi :TQ\rightarrow Q$ with the “magnetic” symplectic form

$$\begin{aligned} \Omega _\epsilon ^* = \pi ^*\omega +\epsilon \,\Omega , \end{aligned}$$

where $\epsilon$ is a real parameter and $\Omega$ is the pullback of the canonical symplectic form on $T^*Q$ along the bundle map $TQ\rightarrow T^*Q$ defined by *g*. We may also define a natural Hamilton function on *TQ*,

$$\begin{aligned} H^*_\epsilon (q,v) =\frac{1}{2} \epsilon ^2\,g_q(v,v) + \epsilon \,H(q). \end{aligned}$$

As explained in detail in Burby and Hirvijoki (2021), $H^*_\epsilon$ defines a Hamiltonian nearly periodic system whose slow manifold dynamics recovers the dynamics of *H* on *Q* as $\epsilon \rightarrow 0$. The limiting roto-rate is

15 $$\begin{aligned} R_0(q,v) = {\mathbb {J}}_q(v-X_H(q))\cdot \partial _v, \end{aligned}$$

where $X_H$ denotes the Hamiltonian vector field on *Q* associated with *H*, and the angular frequency function $\omega _0 = 1$. Moreover, the adiabatic invariant associated with $H^*_\epsilon$ ensures that the slow manifold enjoys long-term normal stability. It is crucial that the metric *g* is determined by an almost complex structure ${\mathbb {J}}$ compatible with $\omega$ for these results to hold. If *g* is a more general metric tensor then the larger system on *TQ* need not be nearly periodic, and an adiabatic invariant need not exist.

The purpose of this section is to combine observations from Burby and Hirvijoki (2021) with the theory of nearly periodic maps in order to construct a geometric numerical integrator for *H*. The integrator will be given as an implicitly defined mapping on *TQ* that is provably presymplectic nearly periodic with limiting roto-rate $R_0$. We will show that this mapping admits a slow manifold diffeomorphic to *Q* on which iterations of the map approximate the *H*-flow. In fact, the mapping is a slow manifold integrator in the sense described in Burby and Klotz (2020). In addition, we will argue using the Noether theorem for nearly periodic maps that this discrete-time slow manifold enjoys long-term normal stability. This ensures that the mapping on *TQ* will function effectively and reliably as a structure-preserving integrator for the original Hamiltonian system on *Q*. We remark that the results described in this section provide a general solution to the problem of structure-preserving integration of non-canonical Hamiltonian systems on exact symplectic manifolds. For a completely different approach that is less geometric, we refer readers to Kraus (2017).

We begin with some preliminary remarks.

#### Remark 4

It will be convenient to work with the parameter $\hbar = \sqrt{\epsilon }$ instead of $\epsilon$. There are technical reasons for doing so that will not be discussed here; however, an obvious physical benefit will be that $\hbar$ may be interpreted as a timestep. The symplectic form on *TQ* will therefore be given by

$$\begin{aligned} \Omega ^*_{\hbar } = \pi ^*\omega + \hbar ^2\,\Omega . \end{aligned}$$

#### Remark 5

It is useful to describe the goal of our construction in more concrete terms. We aim to find a smooth $\hbar$-dependent mapping $\Psi _{\hbar }:TQ\rightarrow TQ$ that is both non-resonant nearly periodic with limiting roto-rate $R_0$ given by (15) and symplectic, $\Psi _{\hbar }^*\Omega ^*_{\hbar } = \Omega ^*_{\hbar }$, for all $\hbar \ll 1$. Since *Q* is connected and $R_0$ has a manifold of fixed points of the form $\{(q,X_H(q))\}\subset TQ$, Theorem 4 (Noether’s theorem for nearly periodic maps) ensures that the mapping we seek will admit an adiabatic invariant $\mu _{\hbar }$.

#### Remark 6

We may determine the leading-order term in the formal power series $\mu _{\hbar } = \mu _0 + \hbar \,\mu _1 +\hbar ^2\,\mu _2 + \dots$ using only the explicit expressions for $\Omega ^*_{\hbar }$ and $R_0$ in conjunction with the general existence theorem (Theorem 4). Recall that the theorem says that the adiabatic invariant is given by $\mu _{\hbar } = \iota _{R_{\hbar }}{\overline{\Theta }}_{\hbar }$, where $R_{\hbar }$ is the roto-rate and ${\overline{\Theta }}_{\hbar }$ is a *U*(1)-invariant primitive for $\Omega ^*_{\hbar }$. In particular, the roto-rate must satisfy Hamilton’s equation $\iota _{R_{\hbar }}\Omega ^*_{\hbar } = {\textbf{d}}\mu _{\hbar }$ with Hamiltonian $\mu _{\hbar }$. We apply this theorem as follows.

If $f:TQ\rightarrow {\mathbb {R}}$ is any smooth $\hbar$-independent function on *TQ* then it is straightforward to verify that $X_f=\hbar ^{-4}({\mathbb {J}}\,\partial _{v}f)\cdot \partial _v+\text {l.o.t.}$. Therefore, we must have $R_{\hbar }=\hbar ^{-4}({\mathbb {J}}\,\partial _{v}\mu _0)\cdot \partial _v+\text {l.o.t.}$. But since $R_{\hbar }=O(1)$, the last expression implies $\partial _v\mu _0=0$ everywhere on *TQ*, or $\mu _0=\mu _0(q)$. In fact $\mu _0(q)$ must be constant, for if $(q,v_{\hbar }(q))$ is a zero for $R_{\hbar }$ then Hamilton’s equation implies $0=\lim _{\hbar \rightarrow 0}{\textbf{d}}\mu _{\hbar }(q,v_{\hbar }(q)) = {\textbf{d}}\mu _0(q)$. And by evaluating the formula $\mu _{\hbar } = \iota _{R_{\hbar }}{\overline{\Theta }}_{\hbar }$ at $(q,v_{\hbar }(q))$ we find that the constant must in fact be 0. In other words $\mu _0 = 0$. Essentially, the same argument may now be applied to conclude $\mu _1=\mu _2=\mu _3=0$; the argument for $\mu _1$ proceeds as follows. Since $\mu _0=0$, Hamilton’s equation implies $R_0 = \hbar ^{-3}({\mathbb {J}}\,\partial _{v}\mu _1)\cdot \partial _v+\text {l.o.t.}$, or $\mu _1 = \mu _1(q)$. Evaluating Hamilton’s equation at $(q,v_{\hbar }(q))$, dividing by $\hbar$, and taking the limit $\hbar \rightarrow 0$ then leads to $0 = \lim _{\hbar \rightarrow 0}\hbar ^{-1}{\textbf{d}}\mu _{\hbar }(q,v_{\hbar }(q)) ={\textbf{d}}\mu _1(q)$, implying $\mu _1$ is a constant. Applying the same procedure to the formula $\mu _{\hbar } = \iota _{R_{\hbar }}{\overline{\Theta }}_{\hbar }$ implies the constant must be 0.

We have now established that the adiabatic invariant for the nearly periodic map we aim to construct must have the form $\mu _{\hbar } =\hbar ^4\,\mu _4 + \hbar ^5\,\mu _5 + \dots$. We can determine an explicit expression for $\mu _4$ as follows. By the above remarks, we must have $R_{\hbar }=({\mathbb {J}}\,\partial _{v}\mu _4)\cdot \partial _v+\text {l.o.t.}$, which implies in particular that $R_0 = ({\mathbb {J}}\,\partial _{v}\mu _4)\cdot \partial _v$. Since the desired form of $R_0$ is known, we therefore obtain the following partial differential equation for $\mu _4$:

$$\begin{aligned} {\mathbb {J}}(v-X_H) = {\mathbb {J}}\,\partial _{v}\mu _4. \end{aligned}$$

The general solution of this equation is given by $\mu _4(q,v) = \frac{1}{2}g_q(v-X_H(q),v-X_H(q))+\chi (q)$, where $\chi (q)$ is an arbitrary function of *q*. To determine $\chi$, we evaluate the formula $\mu _{\hbar } = \iota _{R_{\hbar }}{\overline{\Theta }}_{\hbar }$ at a fixed point $(q,v_{\hbar }(q))$ to find $0 =\lim _{\hbar \rightarrow 0}\hbar ^{-4}\mu _{\hbar }(q,v_{\hbar }(q)) = \mu _4(q,v_0(q))=\chi (q)$. Note that we have used the formula for $v_0(q) = X_H(q)$ for fixed points of $R_0$. We conclude that the adiabatic invariant must have the general form

16 $$\begin{aligned} \mu _{\hbar }(q,v) =\hbar ^{4}\frac{1}{2}g_q(v-X_H(q),v-X_H(q)) + O(\hbar ^{5}). \end{aligned}$$

This formula will be useful later when we argue for long-term normal stability.

To construct the mapping $\Psi _{\hbar }:TQ\rightarrow TQ$, we begin by introducing the mixed-variable generating function

17 $$\begin{aligned} S&:Q\times Q\rightarrow {\mathbb {R}}\nonumber \\ S(q,{\overline{q}})&= \int _q^{{\overline{q}}}\vartheta + \Phi ^*\Sigma (q,{\overline{q}}) , \end{aligned}$$

where $\Phi (q,{\overline{q}}) = (q/2+{\overline{q}}/2,{\overline{q}}-q)$ is a diffeomorphism $Q\times Q\rightarrow TQ$, and $\Sigma :TQ\rightarrow {\mathbb {R}}$ is given by

$$\begin{aligned} \Sigma (x,\xi )&= -\hbar H(x) + \hbar ^2\,\langle X_H(x),\xi \rangle -\frac{1}{12}\hbar ^2 \partial _k\omega _{j\ell }(x)\,X_H^k(x)\,X_H^j(x)\,\xi ^\ell \nonumber \\&\qquad - \frac{1}{4}\left( \frac{\sin \theta _0}{1-\cos \theta _0}\right) \langle \xi -\hbar X_H(x),\xi -\hbar X_H(x) \rangle . \end{aligned}$$

Here, $\langle \cdot ,\cdot \rangle$ is shorthand for $g(\cdot ,\cdot )$, the integral is taken along the straight line connecting *q* with ${\overline{q}}$, $X_H = -{\mathbb {J}}\nabla H$ is the Hamiltonian vector field associated with *H*, and $\theta _0\in \!\!\!\!\!/\{0,\pi \}$. The metric tensor, the Hamiltonian, and the Hamiltonian vector field are evaluated at the midpoint $x=({\overline{q}}+q)/2$. The variables *q* and ${\overline{q}}$ should be interpreted as the “old” and “new” points in the symplectic manifold *Q*. While it is not necessary to go into the details here, the function *S* may be understood as an approximation of Jacobi’s solution of the Hamilton Jacobi equation for the Hamiltonian $H_\epsilon ^*$.

#### Definition 14

The **symplectic Lorentz map** is the mapping $\Psi _{\hbar }:TQ\rightarrow TQ:(q,v)\mapsto ({\overline{q}},{\overline{v}})$ defined by the implicit relations

18 $$\begin{aligned} \vartheta _{{\overline{q}}} + \hbar ^2\,g_{{\overline{q}}}({\overline{v}},d{\overline{q}})&={ {\textbf{d}}^{(2)} }S, \end{aligned}$$

19 $$\begin{aligned} \vartheta _q+ \hbar ^2\,g_{{q}}({v},d{q})&= -{ {\textbf{d}}^{(1)}}S. \end{aligned}$$

Here $g_{{\overline{q}}}({\overline{v}},d{\overline{q}})$ is the linear map $T_{{\overline{q}}}Q\rightarrow {\mathbb {R}}$ given by ${\overline{w}}_{{\overline{q}}}\mapsto g_{{\overline{q}}}({\overline{v}},{\overline{w}}_{{\overline{q}}})$, and $g_{{q}}({v},d{q})$ is defined analogously.

#### Proposition 5

The symplectic Lorentz map is well-defined and smooth in $(q,v,\hbar )$ for $\hbar$ in a neighborhood of $0\in {\mathbb {R}}$. Moreover, it preserves the $\hbar$-dependent symplectic form $\Omega ^*_{\hbar }$ and satisfies

20 $$\begin{aligned} \Psi _0(q,v) = (x,X_H(q)+\exp (-\theta _0\,{\mathbb {J}}(q))[v-X_H(q)]) . \end{aligned}$$

#### Proof

First we will construct a convenient moving frame on $Q\times Q$ onto which we will resolve the implicit relations (18) and (19). We will start by building a frame on *TQ* and then finish by pulling back along the mapping $\Phi :Q\times Q\rightarrow TQ$ defined above. Without loss of generality, assume $Q={\mathbb {R}}^n$ for an even integer *n* and let $(x^i,\xi ^i)$ denote the standard linear coordinate system on *TQ*. Fix $(x,\xi )\in TQ$ and let $\gamma :[-1,1]\rightarrow Q$ be a smooth curve in *Q* with $\gamma (0) = x$. Relative to the Riemannian structure defined by the metric *g* there is a unique horizontal lift ${\tilde{\gamma }}:[-1,1]\rightarrow TQ$ with ${\tilde{\gamma }}(0) = (x,\xi )$. In this manner, to each $(x,\xi )\in TQ$ and each tangent vector $w\in T_xQ$ we assign a lifted tangent vector ${\tilde{w}}\in T_{(x,\xi )}TQ$. Applying this construction point-wise to the coordinate vector fields $\partial _{x^i}$ on *Q*, we obtain linearly independent vector fields ${\tilde{\partial }}_{x^i}$ on *TQ*. The collection of 2*n* vector fields $({\tilde{\partial }}_{x^i},\partial _{\xi ^i})$ comprise a frame on *TQ*. A frame on $Q\times Q$ is then furnished by the vector fields $(U^i,A^i)$, where $U^i = \Phi ^*{\tilde{\partial }}_{x^i}$ and $\quad A^i =\Phi ^*\partial _{\xi ^i}$. Upon introducing the Christoffel symbols $\nabla _{\partial _{x^i}}\partial _{x^j} = \Gamma ^k_{\,\,ij}\,\partial _{x^{k}}$, the vector fields ${\tilde{\partial }}_{x^i}$ may be written as

$$\begin{aligned} {\tilde{\partial }}_{x^i} = \partial _{x^i} -\Gamma ^k_{\,\,ij}(x)\,\xi ^j\,\partial _{\xi ^k}. \end{aligned}$$

Therefore, we may write the following explicit formulas for the frame $(U^i,A^i)$:

$$\begin{aligned} U^i&=\bigg (\partial _{q^i} +\frac{1}{2}\Gamma ^k_{\,\,ij}(x)\,\xi ^j\,\partial _{q^k}\bigg )+ \bigg (\partial _{{\overline{q}}^i} - \frac{1}{2}\Gamma ^k_{\,\,ij}(x)\,\xi ^j\,\partial _{{\overline{q}}^k}\bigg )\\ A^i&= -\frac{1}{2}\partial _{q^i} + \frac{1}{2}\partial _{{\overline{q}}^i}, \end{aligned}$$

where we remind the reader that $x = (q+{\overline{q}})/2$ and $\xi = {\overline{q}}-q$.

Next, we rewrite (18) and (19) as a single equation on $Q\times Q$,

21 $$\begin{aligned} \vartheta _{{\overline{q}}}(d{\overline{q}}) -\vartheta _{q}(dq)+ \hbar ^2\,g_{{\overline{q}}}({\overline{v}},d{\overline{q}}) - \hbar ^2\,g_{{q}}({v},d{q})= {\textbf{d}}S, \end{aligned}$$

and then take components along the frame $(U^i,A^i)$ to obtain

$$\begin{aligned}&\hbar ^2\,g_{\ell \,i}({\overline{q}})\,{\overline{v}}^\ell - \hbar ^2\,g_{\ell \,i}(q)\,v^\ell -\frac{1}{2} \hbar ^2\,\Gamma ^k_{\,\,ij}(x)\,\xi ^j\bigg (g_{\ell \,k}({\overline{q}})\,{\overline{v}}^\ell + g_{\ell \,k}(q)\,v^\ell \bigg )\\&=\int _0^1\xi ^j\omega _{ji}(\lambda ) \,d\lambda -\int _0^1\left[ \lambda -\frac{1}{2}\right] \xi ^j\,\omega _{jk}(\lambda )\Gamma ^k_{\,\,i\ell }(x)\xi ^\ell \,d\lambda \nonumber \\&\qquad -\hbar \,\partial _iH(x) +\frac{\hbar }{2}\left( \frac{\sin \theta _0}{1-\cos \theta _0}\right) \,g_{\ell \,j}(x)\,X^\ell _{H;i}(x)\,(\xi ^j - \hbar \,X_H^j(x))\\&\qquad -\frac{1}{12}\hbar ^2\,\partial _i(\partial _k\omega _{j\ell }\,X_H^k\,X_H^j)\,\xi ^\ell + \frac{1}{12}\hbar ^2\,\partial _k\omega _{j\ell }\,X_H^k\,X_H^j\,\Gamma ^\ell _{\,\,im}\,\xi ^m, \end{aligned}$$

and

$$\begin{aligned}&\frac{1}{2}\,\hbar ^2\,g_{\ell \,i}({\overline{q}})\,{\overline{v}}^\ell + \frac{1}{2}\,\hbar ^2\,g_{\ell \,i}(q)\,v^\ell \\&= \int _0^1\left[ \lambda -\frac{1}{2}\right] \,\xi ^j\omega _{ji}(\lambda )\,d\lambda -\frac{1}{12}\hbar ^2\,\partial _k\omega _{ji}\,X_H^k\,X_H^j \nonumber \\&\qquad + \hbar ^2\,g_{ij}(x)\,X_H^j(x) - \frac{1}{2}\left( \frac{\sin \theta _0}{1-\cos \theta _0}\right) \,g_{ij}(x)(\xi ^j -\hbar \,X_H^j(x)). \end{aligned}$$

Here, we have applied the useful formulas

$$\begin{aligned} {\textbf{d}}\int _{q}^{{\overline{q}}}\vartheta = \vartheta _{{\overline{q}}}(d{\overline{q}}) -\vartheta _q(dq)+\int _0^1\omega _\lambda (\xi ,[1-\lambda ]dq + \lambda \,d{\overline{q}})\,d\lambda \end{aligned}$$

and

$$\begin{aligned} {\mathcal {L}}_{{\tilde{\partial }}_{x^i}}\Sigma&= -\hbar \,\partial _iH +\frac{\hbar }{2}\left( \frac{\sin \theta _0}{1-\cos \theta _0}\right) \,g(\nabla _{\partial _i}X_H,\xi -\hbar \,X_H) \\&\qquad -\frac{1}{12}\hbar ^2\,\partial _i(\partial _k\omega _{j\ell }\,X_H^k\,X_H^j)\,\xi ^\ell + \frac{1}{12}\hbar ^2\,\partial _k\omega _{j\ell }\,X_H^k\,X_H^j\,\Gamma ^\ell _{\,\,im}\,\xi ^m\\ {\mathcal {L}}_{\partial _{\xi ^i}}\Sigma&= \hbar ^2\,g(X_H,\partial _{x^i})-\frac{1}{12}\hbar ^2\,\partial _k\omega _{ji}\,X_H^k\,X_H^j - \frac{1}{2}\left( \frac{\sin \theta _0}{1-\cos \theta _0}\right) \,g(\xi -\hbar \,X_H,\partial _{x^i}), \end{aligned}$$

where $$\omega _\lambda = \omega _{q(\lambda )}$$ with $$q(\lambda ) = [1-\lambda ]\,q + \lambda \,{\overline{q}}$$.

To show that these implicit equations define a smooth $\hbar$-dependent mapping $\Psi _{\hbar }$, we first introduce the new variable $\Delta = \hbar ^{-2}(\xi -\hbar \,X_H(x))$ and then observe that when expressed in terms of $\Delta$ the implicit equations above may be written in the form

$$\begin{aligned}&\hbar ^2\,g_{\ell \,i}(x)\,({\overline{v}}^\ell -v^\ell )=\hbar ^2\Delta ^j\omega _{ji}(x)+O(\hbar ^3) \end{aligned}$$

and

$$\begin{aligned}&\frac{1}{2}\,\hbar ^2\,g_{\ell \,i}(x)\,({\overline{v}}^\ell +v^\ell ) =\hbar ^2\,g_{ij}(x)\,X_H^j(x) - \hbar ^2\frac{1}{2}\left( \frac{\sin \theta _0}{1-\cos \theta _0}\right) \,g_{ij}(x)\Delta ^j+O(\hbar ^3). \end{aligned}$$

Note in particular that the term $\frac{1}{12}\hbar ^2\,\partial _k\omega _{ji}\,X_H^k\,X_H^j$ exactly cancels the second-order part of $\int _0^1\left[ \lambda -\frac{1}{2}\right] \,\xi ^j\omega _{ji}(\lambda )\,d\lambda$. Dividing these expressions by $\hbar ^2$ implies that there are smooth functions $Z_1,Z_2:{\mathbb {R}}^n\times {\mathbb {R}}^n\times {\mathbb {R}}^n\times {\mathbb {R}}^n\times {\mathbb {R}}\rightarrow {\mathbb {R}}^n$ given by

$$\begin{aligned} Z_{1i}(x,v,\Delta ,{\overline{v}},\hbar )&= g_{\ell \,i}(x)\,({\overline{v}}^\ell -v^\ell ) - \Delta ^j\omega _{ji}(x) + O(\hbar ),\\ Z_{2i}(x,v,\Delta ,{\overline{v}},\hbar )&=\frac{1}{2}\,g_{\ell \,i}(x)\,({\overline{v}}^\ell +v^\ell ) - \,g_{ij}(x)\,X_H^j(x) \\&\quad + \frac{1}{2}\left( \frac{\sin \theta _0}{1-\cos \theta _0}\right) \,g_{ij}(x)\Delta ^j+O(\hbar ), \end{aligned}$$

such that the implicit equations defining the symplectic Lorentz map are satisfied if and only if

$$\begin{aligned} Z_1(x,v,\Delta ,{\overline{v}},\hbar ) = Z_2(x,v,\Delta ,{\overline{v}},\hbar ) = 0. \end{aligned}$$

When $\hbar =0$, the unique solution of these equations for $\Delta$ and ${\overline{v}}$ is

$$\begin{aligned} \Delta _0&= (1-\exp (-\theta _0\,{\mathbb {J}}) ){\mathbb {J}}\,(v-X_H(x)),\\ {\overline{v}}_0&= X_H(x) - \exp (-\theta _0\,{\mathbb {J}}) [v - X_H(x)]. \end{aligned}$$

Moreover, for each (*x*, *v*), the $(\Delta ,{\overline{v}})$ derivative of $(Z_1,Z_2)^T$ at $(x,v,\Delta _0,{\overline{v}}_0,0)$ is given by

$$\begin{aligned} \begin{pmatrix} D_{\Delta }Z_1(x,v,\Delta _0,{\overline{v}}_0) &{} D_{{\overline{v}}}Z_1(x,v,\Delta _0,{\overline{v}}_0)\\ D_{\Delta }Z_2(x,v,\Delta _0,{\overline{v}}_0) &{} D_{{\overline{v}}}Z_2(x,v,\Delta _0,{\overline{v}}_0) \end{pmatrix} = \begin{pmatrix} -{\mathbb {J}}(x) &{} 1\\ \frac{1}{2}\left( \frac{\sin \theta _0}{1-\cos \theta _0}\right) &{} \frac{1}{2} \end{pmatrix}, \end{aligned}$$

which is invertible. The implicit function theorem therefore implies there is a unique pair of smooth functions $\Delta (x,v,\hbar ),{\overline{v}}(x,v,\hbar )$ defined in an open neighborhood of $\{(x,v,\hbar )\mid \hbar =0\}\subset {\mathbb {R}}^n\times {\mathbb {R}}^n\times {\mathbb {R}}$ that satisfy the equations

$$\begin{aligned}Z_1(x,v,\Delta (x,v,\hbar ),{\overline{v}}(x,v,\hbar ))=Z_2(x,v,\Delta (x,v,\hbar ),{\overline{v}}(x,v,\hbar ))=0.\end{aligned}$$

Since $\Delta$ is related to ${\overline{q}}$ by

$$\begin{aligned} {\overline{q}} = x +\frac{1}{2} \hbar \,X_H(x) + \frac{1}{2}\hbar ^2\,\Delta (x,v,\hbar ), \end{aligned}$$

another simple application of the implicit function theorem establishes existence and smoothness of the symplectic Lorentz map $\Psi _{\hbar }:(q,v)\mapsto ({\overline{q}},{\overline{v}})$. We have also shown that $\Psi _0$ has the desired form $(q,v)\mapsto (q,{\overline{v}}_0)$. Symplecticity of $\Psi _{\hbar }$ now follows immediately from applying the exterior derivative to (21). $\square$

#### Corollary 2

The symplectic Lorentz map is a presymplectic nearly periodic map. It is non-resonant provided $\theta _0/2\pi \in \!\!\!\!\!/{\mathbb {Q}}$.

We have thus constructed a nearly periodic map with the desired roto-rate and integral invariant. Now we must establish the precise sense in which the symplectic Lorentz map $\Psi _{\hbar }$, which is *a priori* a mapping $TQ\rightarrow TQ$, functions as a consistent numerical integrator for the Hamiltonian system $X_H$ on *Q*. The first hint as to how this might work is that the limit map $\Psi _0$ admits a manifold of fixed points given by $\Gamma _0 = \{(x,v)\in TQ\mid v = X_H(q)\}$. This limiting invariant manifold, being the graph of $X_H$, is manifestly diffeomorphic to *Q*. Thus, if $\Gamma _0$ can be continued to an invariant manifold $\Gamma _{\hbar }$ for $\Psi _{\hbar }$ with $\hbar \ne 0$, we would automatically obtain dynamics on *Q* that could be compared with those of $X_H$ by restricting $\Psi _{\hbar }$ to $\Gamma _{\hbar }$.

Unfortunately, $\Gamma _0$ is unlikely to continue as a true invariant manifold since each fixed point on $\Gamma _0$ is of elliptic type. Instead, we can obtain the following weaker result. Roughly speaking, it says that there is a unique invariant continuation of $\Gamma _0$ at the level of formal power series in $\hbar$.

#### Proposition 6

Denote the components of the symplectic Lorentz map as $\Psi _{\hbar } = (\Psi _{\hbar }^q,\Psi _{\hbar }^v):(q,v)\mapsto ({\overline{q}},{\overline{v}})$. Assume $\theta _0\ne 0\text { mod }2\pi$. There exists a formal power series

$$\begin{aligned} v^*_{\hbar }(q) = X_H(q) + \hbar \,v^*_1(q) + \hbar ^2\,v^*_2(q) + \dots \end{aligned}$$

with vector field coefficients such that

22 $$\begin{aligned} \Psi _{\hbar }^v(q,v^*_{\hbar }(q)) = v^*_{\hbar }(\psi _{\hbar }(q)), \end{aligned}$$

where

$$\begin{aligned} \psi _{\hbar }(q) = \Psi _{\hbar }^q(q,v^*_{\hbar }(q)). \end{aligned}$$

#### Proof

Expanding the condition (22) in powers of $\hbar$ leads to an infinite sequence of constraints that the formal power series $v^*_{\hbar }$ must obey. Simultaneous satisfaction of each constraint in the sequence is equivalent to (22). The first two constraints are given explicitly by

$$\begin{aligned} \Psi _0^{v}(q,X_H(q))&= X_H(q),\\ \Psi _1^v(q,X_H(q)) + D_v\Psi _0^v(q,X_H(q))[v^*_1(q)]&= v^*_1(q) + DX_H(q)[\Psi _{1}^{q}(q,X_H(q))], \end{aligned}$$

where we have introduced the formal series expansions

$$\begin{aligned} \Psi _{\hbar }^q = \Psi ^q_0 + \hbar \,\Psi ^q_1 + \dots ,\quad \Psi _{\hbar }^v = \Psi _{0}^v + \hbar \,\Psi _{1}^v+\dots \end{aligned}$$

and used $\Psi ^q_0(q,v)=q$. Glancing at (20) reveals that the first of these equations is automatically satisfied. The second equation can be interpreted as an algebraic equation constraining the form of $v^*_1$. In fact, since the linear map

$$\begin{aligned} L(q) = D_v\Psi _0^v(q,X_H(q)) - \text {id},\\ \delta v\mapsto \exp (-\theta _0\,{\mathbb {J}}(q))[\delta v] - \delta v, \end{aligned}$$

is invertible whenever $\theta _0\ne 0\text { mod }2\pi$, $v^*_1$ is determined uniquely by the formula

$$\begin{aligned} v^*_1(q) = L(q)^{-1}[DX_H(q)[\Psi _{1}^{q}(q,X_H(q))] - \Psi _1^v(q,X_H(q)) ]. \end{aligned}$$

More generally, the $n^{\text {th}}$ equation in the sequence has the form

$$\begin{aligned} L(q)[v_n^*(q)] = s_n(q), \end{aligned}$$

where $s_n(q)$ depends only on coefficients of the power series expansion for $\Psi _{\hbar }$ and coefficients $v^*_k$ with $k<n$. Invertibility of *L*(*q*) therefore implies that there is a unique formula for $v^*_n$ for each *n*. The formal power series $v^*_{\hbar }$ defined in this manner satisfies (22) by construction. $\square$

So while we do not obtain a genuine invariant manifold diffeomorphic to *Q*, we do obtain a family of approximate invariant manifolds diffeomorphic to *Q* given by truncations of the formal power series $v^*_{\hbar }$. Using arguments comparable to those presented in Burby and Hirvijoki (2021), it is possible to show that truncations of $v^*_{\hbar }$ may be constructed so their graphs agree with the zero level set of the adiabatic invariant $\mu _{\hbar }$ for $\Psi _{\hbar }$ to any desired order in $\hbar$. Adiabatic invariance of $\mu _{\hbar }$ can then be used to prove the existence of manifolds $\Gamma _{\hbar }^{(n)}$ close to $\Gamma _0$ with the following schematic normal stability property:

- For each $N>0$ and (*q*, *v*) within $\hbar ^{\alpha (n)}$ of $\Gamma _{\hbar }^{(n)}$ the point $\Psi _{\hbar }^{k}(q,v)$ remains within $\hbar ^{\beta (n)}$ of $\Gamma _{\hbar }^{(n)}$ for $k=O(\epsilon ^{-N})$. Here $\alpha$ and $\beta$ are monotone increasing functions of *n*.

We will not attempt to prove such a result in full generality here. However, since we have already determined the form of the leading term in the adiabatic invariant series (in this case $\mu _{\hbar } = \hbar ^4\,\mu _4 + O(\hbar ^5)$), we can prove a special case of the general result without much effort. First we establish the timescale over which $\mu _4$ is well-conserved.

#### Definition 15

Given a compact set $C\subset TQ$, a point (*q*, *v*) is **positively contained** if $\Psi _{\hbar }^k(q,v)\in C$ for all nonnegative integers *k* and all $\hbar$ in a neighborhood of 0.

#### Proposition 7

For each $N>0$ and compact set $C\subset TQ$ there is a positive, $\hbar$-independent constant ${\mathcal {M}}$ such that

$$\begin{aligned} |\mu _4(\Psi _{\hbar }^k(q,v))-\mu _4(q,v)|\le {\mathcal {M}}\,\hbar ,\quad k\in [0,k^*(\hbar ,N)], \end{aligned}$$

whenever (*q*, *v*) is positively contained in *C*. Here $k^*(\hbar ,N) = O(\hbar ^{-N})$.

#### Proof

First we will obtain a useful estimate for the degree of conservation of an arbitrary truncation of the adiabatic invariant series. Let ${\overline{\mu }}_{\hbar } = \hbar ^{-4}\mu _{\hbar } = \mu _4 + \hbar \,\mu _5 + \dots$ denote the reduced adiabatic invariant for the symplectic Lorentz map. Define ${\overline{\mu }}_i = \mu _{i+4}$ and let ${\overline{\mu }}_{\hbar }^{(N)} = \sum _{i=0}^{N}{\overline{\mu }}_i\,\hbar ^{i}$. Since ${\overline{\mu }}_{\hbar }^{(N)} = {\overline{\mu }}_{\hbar } + O(\hbar ^{N+1})$ and ${\overline{\mu }}_{\hbar }$ is $\Psi _{\hbar }$-invariant to all orders in $\hbar$, there is a constant ${\mathcal {M}}_N$, depending on both *C* and *N*, such that

$$\begin{aligned} \forall (q,v)\in C,\,|{\overline{\mu }}_{\hbar }^{(N)}(\Psi _{\hbar }(q,v)) - {\overline{\mu }}_{\hbar }^{(N)}(q,v) | \le {\mathcal {M}}_N\,\hbar ^{N+1} . \end{aligned}$$

For positively contained (*q*, *v*), we may apply this formula repeatedly to obtain an estimate for the change in ${\overline{\mu }}_{\hbar }^{(N)}$ after *k* positive timesteps,

23 $$\begin{aligned} |{\overline{\mu }}_{\hbar }^{(N)}(\Psi _{\hbar }^k(q,v)) - {\overline{\mu }}_{\hbar }^{(N)}(q,v) |&\le {\mathcal {M}}_{N}\,\hbar ^{N+1} + |{\overline{\mu }}_{\hbar }^{(N)}(\Psi _{\hbar }^{k-1}(q,v)) - {\overline{\mu }}_{\hbar }^{(N)}(q,v) |\nonumber \\&\le (1+k){\mathcal {M}}_N\,\hbar ^{N+1}. \end{aligned}$$

Next, we draw implications from the previous inequality together with a bound on the difference between ${\overline{\mu }}_{\hbar }^{(0)} = \mu _4$ and ${\overline{\mu }}_{\hbar }^{(N)}$. There must be another positive constant ${\mathcal {M}}^\prime _{N}$, depending on both *C* and *N*, such that

$$\begin{aligned} \forall (q,v)\in C,\,|{\overline{\mu }}_{\hbar }^{(0)}(q,v) - {\overline{\mu }}_{\hbar }^{(N)}(q,v) | \le {\mathcal {M}}^\prime _N\,\hbar . \end{aligned}$$

In light of the inequality (23), this implies that for each positively contained (*q*, *v*) the change in ${\overline{\mu }}_{\hbar }^{(0)}$ after *k* positive timesteps is at most

$$\begin{aligned}&|{\overline{\mu }}_{\hbar }^{(0)}(\Psi _{\hbar }^k(q,v)) - {\overline{\mu }}_{\hbar }^{(0)}(q,v) |\\&\quad \le |{\overline{\mu }}_{\hbar }^{(0)}(\Psi _{\hbar }^k(q,v)) - {\overline{\mu }}_{\hbar }^{(N)}(\Psi ^k_{\hbar }(q,v)) | + | {\overline{\mu }}_{\hbar }^{(N)}(\Psi ^k_{\hbar }(q,v))- {\overline{\mu }}_{\hbar }^{(0)}(q,v) |\\&\quad \le {\mathcal {M}}^\prime _{N}\,\hbar + | {\overline{\mu }}_{\hbar }^{(N)}(\Psi _{\hbar }^k(q,v))- {\overline{\mu }}_{\hbar }^{(0)}(q,v) |\\&\quad \le {\mathcal {M}}^\prime _{N}\,\hbar + | {\overline{\mu }}_{\hbar }^{(N)}(\Psi ^k_{\hbar }(q,v))- {\overline{\mu }}_{\hbar }^{(N)}(q,v) | \\&\qquad + |{\overline{\mu }}_{\hbar }^{(N)}(q,v)- {\overline{\mu }}_{\hbar }^{(0)}(q,v) |\\&\quad \le 2{\mathcal {M}}^\prime _{N}\,\hbar + (1+k){\mathcal {M}}_N\,\hbar ^{N+1}. \end{aligned}$$

Apparently, the change in $\mu _4 = {\overline{\mu }}_{\hbar }^{(0)}$ is at most $O(\hbar )$ as long as $k\,\hbar ^{N+1}=O(\hbar )$. We therefore obtain the desired inequality with $k^*(\hbar ,N) = \lfloor {\hbar }^{-N}\rfloor -1$.

$$\square $$

#### Remark 7

If “positively contained” fails, then the next best thing would be having uniform bounds (in *TQ* and $\hbar$) on the derivatives of $\Psi _{\hbar }$. The precise form of these bounds would depend on the details of the underlying Hamiltonian system on *Q*. Then, the proof of the previous proposition would go through practically unchanged. In the absence of both positively containment and uniform boundedness, things get trickier, and we have no general answer as to the validity of Proposition 7.

Using this result together with the explicit form of $\mu _4$, we now easily obtain the following normal stability result for the almost invariant set given by the graph of $X_H$.

#### Proposition 8

Let $C\subset TQ$ be a compact set and set $(q^k,v^k)=\Psi _{\hbar }^k(q,v)$ for any $(q,v)\in TQ$. Let $|\cdot |$ denote the velocity norm provided by the metric tensor *g*. For each $N > 0$, $V_0>0$, and positively contained $(q,v)\in C$ that satisfies

$$\begin{aligned} |v-X_H(q)|_q<V_0\,\sqrt{\hbar }, \end{aligned}$$

there is a positive constant $V_1$ such that

$$\begin{aligned} |v^k-X_H(q^k)|_{q^k}\le V_1\,\sqrt{\hbar } \end{aligned}$$

for all $k\in [0,k^*(\hbar ,N)]$. Here, $k^*(\hbar ,N) = O(\hbar ^{-N})$.

#### Proof

Let $(q,v)\in C$ be positively contained and suppose $|v-X_H(q)|_q<V_0\,\hbar$. By Proposition 7, we have

$$\begin{aligned} |\mu _4(\Psi _{\hbar }^k(q,v))-\mu _4(q,v)|\le {\mathcal {M}}\,\hbar , \end{aligned}$$

for some *N*-dependent constant ${\mathcal {M}}$ and $k\in [0,k^*(\hbar ,N)]$. But since $\mu _4(q,v) =\frac{1}{2} |v-X_H(q)|_q^2$ we can apply this inequality to obtain

$$\begin{aligned} |v^k-X_H(q^k)|_{q^k}^2&\le ||v^k-X_H(q^k)|_{q^k}^2 - |v-X_H(q)|_q^2| + |v-X_H(q)|_q^2\\&\le 2|\mu _4(\Psi _{\hbar }^k(q,v))-\mu _4(q,v)| + V_0^2\,\hbar \\&\le 2{\mathcal {M}}\,\hbar + V_0^2\,\hbar . \end{aligned}$$

Taking a square root gives the desired result. $\square$

In the above sense, the graph of $X_H$ behaves much like a true invariant set over very large time intervals. Of course, the invariance need not be exact, but may include oscillations around the graph of amplitude $\sqrt{\hbar }$. The amplitude of these oscillations can be reduced by considering manifolds that better approximate the zero level set of $\mu _{\hbar }$, but, as mentioned earlier, we will not pursue this matter further in this article.

To complete the picture of how the symplectic Lorentz map may be used as an integrator for $X_H$ on *Q*, we will now describe the precise sense in which the map’s dynamics approximate the *H*-flow. We start with a simple estimate that says the *q*-component of the symplectic Lorentz map approximates the time-$\hbar$ flow of $X_H$ on *Q* with an $O(\hbar ^{5/2})$ error, *provided* the map is applied in an $O(\hbar ^{1/2})$ neighborhood of the graph $\{v=X_H(q)\}$.

#### Proposition 9

Let $(q,v_{\hbar })$ be a smooth $\hbar$-dependent point in *TQ* with $v_{\hbar } = X_H(q) + O(\hbar ^{1/2})$. The mapped point $({\overline{q}},{\overline{v}})=\Psi _{\hbar }(q,v_{\hbar })$ satisfies

$$\begin{aligned} {\overline{q}}&= q + \hbar \,X_H(q) +\frac{1}{2}\hbar ^2\,DX_H(q)[X_H(q)] + O(\hbar ^{5/2}),\\ {\overline{v}}&=X_H({\overline{q}})+O(\hbar ^{1/2}). \end{aligned}$$

#### Proof

In the proof of Proposition 5, we already established

$$\begin{aligned} {\overline{q}}&= x +\frac{1}{2} \hbar \,X_H(x) + \frac{1}{2}\hbar ^2\,\Delta (x,v_{\hbar },\hbar ),\\ {\overline{v}}&= X_H(x) - \exp (-\theta _0\,{\mathbb {J}}_x) [v_{\hbar } - X_H(x)]+O(\hbar ), \end{aligned}$$

and

$$\begin{aligned} \Delta (x,v_{\hbar },0)&= (1-\exp (-\theta _0\,{\mathbb {J}}) ){\mathbb {J}}\,(v_{\hbar }-X_H(x)), \end{aligned}$$

where $x= {\overline{q}}/2 + q/2$. Implicit differentiation of these formulas together with Taylor’s theorem with remainder therefore implies

$$\begin{aligned} {\overline{q}}&= q + \hbar \,X_H(q)+\frac{1}{2}\hbar ^2\,DX_H(q)[X_H(q)] + \hbar ^2\,(1-\exp (-\theta _0\,{\mathbb {J}}_q) ){\mathbb {J}}_q\,\\&\quad (v_{\hbar }-X_H(q)) + O(\hbar ^3),\\ {\overline{v}}&= X_H(q) - \exp (-\theta _0\,{\mathbb {J}}_q) [v_{\hbar } - X_H(q)]+O(\hbar ) \end{aligned}$$

The desired result now follows immediately from $v_{\hbar }-X_H(q) = O(\hbar ^{1/2})$. $\square$

Combining this result with our earlier estimate of the normal stability timescale for $\{v=X_H(q)\}$ in Proposition 8 finally allows us to conclude that the *q*-component of the symplectic Lorentz map provides a persistent approximation of the *H*-flow over very large time intervals provided initial conditions are chosen close enough to the graph $\{v=X_H(q)\}$.

#### Corollary 3

(Persistent approximation property) Let *C* be a compact set and let $(q,v_{\hbar })\in C$ be a smooth $\hbar$-dependent point in *C* that is positively contained for each $\hbar$. Also assume $v_{\hbar } = X_H(q) + O(\hbar ^{1/2})$. For each $N>0$ there is an integer $k^*(\hbar ,N)=O(\hbar ^{-N})$ such that

$$\begin{aligned} q^{k+1}&= q^k + \hbar \,X_H(q^k) +\frac{1}{2}\hbar ^2\,DX_H(q^k)[X_H(q^k)] + O(\hbar ^{5/2}),\\ v^{k+1}&=X_H(q^{k+1})+O(\hbar ^{1/2}), \end{aligned}$$

for each $k\in [0,k^*(\hbar ,N)]$. Here, $(q^k,v^k) = \Psi _{\hbar }^k(q,v_{\hbar })$.

#### Proof

Proposition 8 ensures that the iterates $(q^k,v^k)$ remain within $O(\hbar ^{1/2})$ of $\{v=X_H(q)\}$ for *k* in the desired range. Thus, Proposition 9 applies to each iterate individually, which is precisely the desired result. $\square$

In summary, we have established the following remarkable properties of the symplectic Lorentz map $\Psi _{\hbar }$.

1. It is symplectic on *TQ*, when *TQ* is endowed with the magnetic symplectic form $\Omega ^*_{\hbar } = \pi ^*\omega + \hbar ^2\,\Omega$.
2. Its *q*-component provides an approximation of the time-$\hbar$ flow of $X_H$ with $O(\hbar ^{5/2})$ local truncation error when applied to points in *TQ* within $O(\hbar ^{1/2})$ of $\{v=X_H(q)\}$.
3. If an initial condition is chosen to lie within $\hbar ^{1/2}$ of $\{v=X_H(q)\}$ then it will remain within $\hbar ^{1/2}$ of the same set for a number of iterations that scales like $\hbar ^{-N}$ for any *N*.

## Examples

### Hidden-Variable Newtonian Gravity

In this section, we will use nearly periodic maps to construct a discrete-time model of Newtonian gravitation where the gravitational constant has a dynamical origin. Let $M = {\mathbb {R}}\times {\mathbb {R}}\times {\mathbb {R}}^{d}\times {\mathbb {R}}^d\ni (q,p,{\varvec{Q}},{\varvec{P}})$ and set $\Omega _\gamma = dq\wedge dp + \sum _{i=1}^d dQ^i\wedge dP_i$. Let $V,W:{\mathbb {R}}^d\rightarrow {\mathbb {R}}$ be smooth functions. The Hamiltonian

24 $$\begin{aligned} H_\epsilon (q,p,{\varvec{Q}},{\varvec{P}})&= \frac{1}{2}\,(p^2 + q^2) + \epsilon \,\bigg (\frac{1}{2}|{\varvec{P}}|^2+V({\varvec{Q}}) +q^2\,W({\varvec{Q}})\bigg ) \end{aligned}$$

defines a continuous-time nearly periodic Hamiltonian system with equations of motion

25 $$\begin{aligned} {\dot{p}}&= -q - \epsilon \,2\,q\,W({\varvec{Q}}) \end{aligned}$$

26 $$\begin{aligned} {\dot{q}}&= p \end{aligned}$$

27 $$\begin{aligned} \dot{{\varvec{P}}}&= -{\epsilon }\,\partial _{{\varvec{Q}}}V- \epsilon \, q^2\, \partial _{{\varvec{Q}}}W \end{aligned}$$

28 $$\begin{aligned} \dot{{\varvec{Q}}}&= \epsilon \,{\varvec{P}}. \end{aligned}$$

The angular frequency function is $\omega _0 = 1$, the limiting roto-rate is $R_0 = -q\,\partial _p + p\,\partial _q$, and the corresponding *U*(1)-action is $\Phi _\theta (q,p,{\varvec{Q}},{\varvec{P}}) = (\cos \theta \,q + \sin \theta \,p,\cos \theta \,p-\sin \theta \,q,{\varvec{Q}},{\varvec{P}})$. When $\epsilon =0$, the system’s flow is $F_t(q,p,{\varvec{Q}},{\varvec{P}}) = \Phi _t(q,p,{\varvec{Q}},{\varvec{P}})$. Intuitively, the (*q*, *p*) variables correspond to a fast oscillator that couples nonlinearly to a mechanical system parameterized by $({\varvec{Q}},{\varvec{P}})$. The averaged Hamiltonian for the coupled system is

29 $$\begin{aligned} \frac{1}{2\pi }\int _0^{2\pi }\Phi _\theta ^*H_\epsilon \,d\theta = \mu _0+\epsilon \,\bigg (\frac{1}{2}|{\varvec{P}}|^2+V({\varvec{Q}}) +\mu _0\,W({\varvec{Q}})\bigg ), \end{aligned}$$

where $\mu _0 = \frac{1}{2}(p^2+q^2)$ is the leading-order adiabatic invariant. We therefore expect the slow variables $({\varvec{Q}},{\varvec{P}})$ to behave like a particle in *d*-dimensional space subject to the effective potential $V({\varvec{Q}})+ \mu _0\,W({\varvec{Q}})$.

We will construct a Hamiltonian non-resonant nearly periodic map that accurately simulates the slow dynamics for this system while “stepping over” the shortest scale $2\pi /\omega _0\sim 1$. If $h\in {\mathbb {R}}$ denotes the temporal step size, these requirements translate into symbols as $1\ll h\ll \epsilon ^{-1}$. Upon introducing the parameters $\delta = 1/h$, $\hbar = \epsilon \,h$, and $\gamma = (\hbar ,\delta )$, we may state our requirement equivalently as $|\gamma |\ll 1$. Our construction will now proceed using the method of mixed-variable generating functions.

The exact Type I generating function for this problem can be characterized by Jacobi’s solution of the Hamilton–Jacobi equation, which is given by

30 $$\begin{aligned} S(q,{\varvec{Q}},{\overline{q}},\overline{{\varvec{Q}}})=\int _0^h \left[ p(t){\dot{q}}(t) + {\varvec{P}}(t)\dot{{\varvec{Q}}}(t)-H(q(t),{\varvec{Q}}(t),p(t),{\varvec{P}}(t))\right] dt, \end{aligned}$$

where $(q(t),{\varvec{Q}}(t),p(t),{\varvec{P}}(t))$ satisfies Hamilton’s equations, and the boundary conditions $q(0)=q$, ${\varvec{Q}}(0)={\varvec{Q}}$, $q(h)={\overline{q}}$, ${\varvec{Q}}(h)=\overline{{\varvec{Q}}}$. In the setting of variational integrators (Marsden and West 2001), this is referred to as the exact discrete Lagrangian, and there are also exact discrete Hamiltonians (Leok and Zhang 2011) corresponding to Type II and Type III generating functions. One possible way to construct a computable approximation of the exact Type I generating function is to observe that it can also be expressed as

31 $$\begin{aligned}&S(q,{\varvec{Q}},{\overline{q}},\overline{{\varvec{Q}}})={{\,\textrm{ext}\,}}_{\begin{array}{c} (q, p, {\varvec{Q}},{\varvec{P}}) \in C^2([0, h],M)\\ q(0)=q, q(h)={\overline{q}},\\ {\varvec{Q}}(0)={\varvec{Q}}, {\varvec{Q}}(h)=\overline{{\varvec{Q}}} \end{array}}\nonumber \\&\quad \int _0^h \left[ p(t){\dot{q}}(t) + {\varvec{P}}(t)\dot{{\varvec{Q}}}(t)-H(q(t),{\varvec{Q}}(t),p(t),{\varvec{P}}(t))\right] dt, \end{aligned}$$

Then, one can construct a computable approximation by replacing the infinite-dimensional function space $C^2([0, h],M)$ with a finite-dimensional subspace, and replacing the integral with a numerical quadrature formula, which yields a Galerkin discrete Lagrangian. Under a number of technical assumptions, the resulting variational integrators $\Gamma$-converge to the exact flow map (Müller and Ortiz 2004), and a quasi-optimality result (Hall and Leok 2015) implies that the rate of convergence is related to the best approximation properties of the finite-dimensional function space used to construct the Galerkin discrete Lagrangian. In general, this means that a good integrator can be constructed by choosing a finite-dimensional function space that is rich enough to approximate the exact solutions well, and using a quadrature rule that is accurate for that choice of function space.

This might entail augmenting the function space with the solution of the fast dynamics when the slow variables are frozen, and then using a quadrature rule that is well adapted to highly oscillatory integrals, like Filon quadrature (Iserles and Nørsett 2004). In this case, the problem exhibits a fast–slow structure that lends itself to a hybrid approximation. We exploit the timescale separation to approximate the fast variables of the dynamics (*q*(*t*), *p*(*t*)) by the exact solution of the $\epsilon =0$ limiting system,

$$\begin{aligned} {\dot{p}} = -q, \quad {\dot{q}} = p, \quad \dot{{\varvec{P}}} =0, \quad \dot{{\varvec{Q}}} = 0. \end{aligned}$$

where the slow variables $({\varvec{Q}}(t),{\varvec{P}}(t))$ are frozen, which leads to a sinusoidal solution for (*q*, *p*). Furthermore, because the timestep *h* is assumed to be large enough that the fast variables perform many revolutions in that time, we anti-alias the fast dynamics by replacing the revolutions by just the fractional part of the revolutions, which we denote by $\theta _0$, and which is assumed to be some irrational multiple of $2\pi$, so that the invariant distribution remains the same. The component of the action integral associated with the fast variables can be evaluated analytically in this case. As for the slow variables, we adopt an approach that can be used to derive the implicit midpoint rule, which is a symplectic integrator for Hamiltonian systems. This involves approximating the solution space by linear functions, so ${\varvec{Q}}(t)$ is uniquely determined by the boundary conditions, and approximating the integral by the midpoint rule. The use of mixed quadrature approximations of the action integral was the basis for implicit–explicit variational integrators for fast–slow systems (Stern and Grinspun 2009).

Note that $\omega _0=1$, then for the (*q*, *p*) dynamics to have a $\theta _0$ rotation in time *h*, the solution is given by

$$\begin{aligned} \begin{bmatrix} q(t) \\ p(t) \end{bmatrix} = \begin{bmatrix} \cos (t) &{} \sin (t)\\ -\sin (t) &{} \cos (t) \end{bmatrix} \begin{bmatrix} q \\ p \end{bmatrix}, \end{aligned}$$

where $\theta _0/h=1$. Since $q(t)=\sin (t) p + \cos (t) q$, then ${\overline{q}}=q(h)=\sin (\theta _0) p + \cos (\theta _0) q$, and hence, $p=\frac{{\overline{q}}-\cos (\theta _0) q}{\sin (\theta _0)}$. Therefore, the (*q*, *p*) dynamics, expressed in terms of the boundary data, is given by

$$\begin{aligned} \begin{bmatrix} q(t) \\ p(t) \end{bmatrix} = \begin{bmatrix} \cos (t) &{} \sin (t)\\ -\sin (t) &{} \cos (t) \end{bmatrix} \begin{bmatrix} q\\ \frac{{\overline{q}}-\cos (\theta _0) q}{\sin (\theta _0)} \end{bmatrix} =\begin{bmatrix} \cos (t) q+\sin (t) \frac{{\overline{q}}-\cos (\theta _0) q}{\sin (\theta _0)}\\ -\sin (t) q+\cos (t) \frac{{\overline{q}}-\cos (\theta _0) q}{\sin (\theta _0)} \end{bmatrix}. \end{aligned}$$

For the slow degrees of freedom, we consider a linear interpolant in time, ${\varvec{Q}}(t)={\varvec{Q}}+\frac{\overline{{\varvec{Q}}}-{\varvec{Q}}}{h}t$, from which the momentum becomes ${\varvec{P}}(t)=\frac{1}{\epsilon }\dot{{\varvec{Q}}}(t)=\frac{1}{\epsilon }\frac{\overline{{\varvec{Q}}}-{\varvec{Q}}}{h}$. Collecting all of these, we have

$$\begin{aligned} q(t)= & {} \cos (t) q+\sin (t) \frac{{\overline{q}}-\cos (\theta _0) q}{\sin (\theta _0)}, {\varvec{Q}}(t)={\varvec{Q}}+\frac{\overline{{\varvec{Q}}}-{\varvec{Q}}}{h}t,\\ {\dot{q}}(t)= & {} -\sin (t) q+\cos (t) \frac{{\overline{q}}-\cos (\theta _0) q}{\sin (\theta _0)}, \dot{{\varvec{Q}}}(t)= \frac{\overline{{\varvec{Q}}}-{\varvec{Q}}}{h},\\ p(t)= & {} -\sin (t) q+\cos (t) \frac{{\overline{q}}-\cos (\theta _0) q}{\sin (\theta _0)}, {\varvec{P}}(t)=\frac{1}{\epsilon }\frac{\overline{{\varvec{Q}}}-{\varvec{Q}}}{h}. \end{aligned}$$

With these, we are now ready to approximate the exact Type I generating function,

$$\begin{aligned} S(q,{\varvec{Q}},{\overline{q}},\overline{{\varvec{Q}}})&=\int _0^h \left[ p(t){\dot{q}}(t) + {\varvec{P}}(t)\dot{{\varvec{Q}}}(t)-H(q(t),{\varvec{Q}}(t),p(t),{\varvec{P}}(t))\right] dt\\&=\int _0^h \Bigg [ p(t){\dot{q}}(t) -\frac{1}{2}\,(p(t)^2 + q(t)^2)\\&\qquad + {\varvec{P}}(t)\dot{{\varvec{Q}}}(t)-\epsilon \,\bigg (\frac{1}{2}|{\varvec{P}}(t)|^2+V({\varvec{Q}}(t)) +q(t)^2\,W({\varvec{Q}}(t))\bigg )\Bigg ] dt, \end{aligned}$$

where we observe that the integral involving the (*q*, *p*) terms has the form of an trigonometric integral, which can be evaluated analytically,

$$\begin{aligned}&\int _0^h \left[ p(t){\dot{q}}(t)-\frac{1}{2}\,(p^2 + q^2)\right] \\&\qquad = \int _0^h \left[ (p^2\cos ^2(t)-2pq\cos (t)\sin (t)+q^2\sin ^2(t))-\frac{1}{2}(p^2+q^2)\right] dt\\&\qquad =\int _0^h \left[ \left( p^2\left( \frac{1+\cos (2t)}{2}\right) -pq\sin (2t)+q^2\left( \frac{1-\cos (2t)}{2}\right) \right) -\frac{1}{2}(p^2+q^2)\right] dt\\&\qquad =\int _0^h \left[ \left( \frac{1}{2}(p^2-q^2)\cos (2t)-pq\sin (2t)\right) \right] dt\\&\qquad =\left. \frac{1}{4}(p^2-q^2)\sin (2t)+\frac{1}{2}pq\cos (2t)\right| _0^{\theta _0}\\&\qquad =\frac{\cos (\theta _0)\tfrac{1}{2}{\overline{q}}^2+\cos (\theta _0)\tfrac{1}{2}q^2-q{\overline{q}}}{\sin (\theta _0)} \end{aligned}$$

where we made use of the trigonometric double angle formulas, $p=\frac{{\overline{q}}-\cos (\theta _0) q}{\sin (\theta _0)}$, and $h=\theta _0$. It is easy to verify that this generating function generates a $\theta _0$ rotation in the (*q*, *p*) variables.

We approximate the integral involving the $({\varvec{Q}},{\varvec{P}})$ terms with the midpoint rule,

$$\begin{aligned}&\int _0^h \left[ {\varvec{P}}(t)\dot{{\varvec{Q}}}(t) - \epsilon \,\bigg (\frac{1}{2}|{\varvec{P}}(t)|^2+V({\varvec{Q}}(t)) +q(t)^2\,W({\varvec{Q}}(t))\bigg )\right] dt\\&\qquad \approx h\left[ \frac{1}{\epsilon }\left( \frac{\overline{{\varvec{Q}}}-{\varvec{Q}}}{h}\right) ^2-\epsilon \left( \frac{1}{2}\frac{1}{\epsilon ^2}\left( \frac{\overline{{\varvec{Q}}}-{\varvec{Q}}}{h}\right) ^2+V\left( \frac{{\varvec{Q}}+\overline{{\varvec{Q}}}}{2}\right) \right. \right. \\&\quad \qquad \left. \left. +q\left( \frac{h}{2}\right) ^2W\left( \frac{{\varvec{Q}}+\overline{{\varvec{Q}}}}{2}\right) \right) \right] \\&\qquad = h\left[ \frac{1}{2\epsilon }\left( \frac{\overline{{\varvec{Q}}}-{\varvec{Q}}}{h}\right) ^2-\epsilon \left( V\left( \frac{{\varvec{Q}}+\overline{{\varvec{Q}}}}{2}\right) +q\left( \frac{h}{2}\right) ^2W\left( \frac{{\varvec{Q}}+\overline{{\varvec{Q}}}}{2}\right) \right) \right] . \end{aligned}$$

By using $\hbar =\epsilon h$, replacing $q\left( \frac{h}{2}\right)$ with $q(0)=q$, and combining it with the first term coming from the fast dynamics, we obtain the following Type I generating function:

32 $$\begin{aligned} S_\gamma (q,{\varvec{Q}},{\overline{q}},\overline{{\varvec{Q}}})&= \frac{\cos \theta _0\,\tfrac{1}{2}q^2 + \cos \theta _0\tfrac{1}{2}{\overline{q}}^2 - q{\overline{q}}}{\sin \theta _0} +\frac{1}{2}\frac{|\overline{{\varvec{Q}}}-{{\varvec{Q}}}|^2}{\hbar }\nonumber \\&\qquad -\hbar \,V\left( \frac{{\varvec{Q}}+\overline{{\varvec{Q}}}}{2}\right) - \hbar \, q^2 W\left( \frac{{\varvec{Q}}+\overline{{\varvec{Q}}}}{2}\right) , \end{aligned}$$

where $\theta _0$ is some irrational multiple of $2\pi$. The implicit relations

33 $$\begin{aligned} {\overline{p}}=\partial _{{\overline{q}}}S_\gamma ,\quad p = -\partial _{q}S_\gamma ,\quad \overline{{\varvec{P}}} = \partial _{\overline{{\varvec{Q}}}}S_\gamma ,\quad {\varvec{P}} = -\partial _{{\varvec{Q}}}S_\gamma , \end{aligned}$$

define a $\gamma$-dependent symplectic map $F_\gamma :(q,p,{\varvec{Q}},{\varvec{P}})\mapsto ({\overline{q}},{\overline{p}},\overline{{\varvec{Q}}},\overline{{\varvec{P}}})$. For small $\gamma$, we claim this map accurately captures the averaged dynamics of the slow variables in the system (25)–(28) and preserves the adiabatic invariant $\mu _0$ over very large time intervals. To show this, we first compute the derivatives in (33) to explicitly write the defining equations for $F_\gamma$ as

$$\begin{aligned} {\overline{p}}&= \frac{\cos \theta _0}{\sin \theta _0}{\overline{q}} - \frac{1}{\sin \theta _0}q, \\ p&= -\frac{\cos \theta _0}{\sin \theta _0}q + \frac{1}{\sin \theta _0}{\overline{q}} + \hbar \,2q\,W\left( \frac{{\varvec{Q}}+\overline{{\varvec{Q}}}}{2}\right) ,\\ \overline{{\varvec{P}}}&= \frac{\overline{{\varvec{Q}}}-{\varvec{Q}}}{\hbar }-\frac{1}{2}\hbar \,V^\prime \left( \frac{{\varvec{Q}}+\overline{{\varvec{Q}}}}{2}\right) - \frac{1}{2}\hbar \,q^2\, W^\prime \left( \frac{{\varvec{Q}}+\overline{{\varvec{Q}}}}{2}\right) ,\\ {\varvec{P}}&= \frac{\overline{{\varvec{Q}}}-{\varvec{Q}}}{\hbar } +\frac{1}{2}\hbar \,V^\prime \left( \frac{{\varvec{Q}}+\overline{{\varvec{Q}}}}{2}\right) + \frac{1}{2}\hbar \,q^2\,W^\prime \left( \frac{{\varvec{Q}}+\overline{{\varvec{Q}}}}{2}\right) . \end{aligned}$$

The first pair of equations can be solved explicitly for ${\overline{p}}$ and ${\overline{q}}$, giving

34 $$\begin{aligned} {\overline{p}}&= \cos \theta _0\,p - \sin \theta _0\,q - \cos \theta _0\,\hbar \,2q\,W\left( \frac{{\varvec{Q}}+\overline{{\varvec{Q}}}}{2}\right) , \end{aligned}$$

35 $$\begin{aligned} {\overline{q}}&= \cos \theta _0\,q + \sin \theta _0\,p - {\sin \theta _0}\, \hbar \,2q\,W\left( \frac{{\varvec{Q}}+\overline{{\varvec{Q}}}}{2}\right) . \end{aligned}$$

Adding and subtracting the last ${\varvec{P}}$ and $\overline{{\varvec{P}}}$ equations then gives

36 $$\begin{aligned} \overline{{\varvec{P}}} - {\varvec{P}}&= -\hbar \, V^\prime \left( \frac{{\varvec{Q}}+\overline{{\varvec{Q}}}}{2}\right) - \hbar \,q^2\,W^\prime \left( \frac{{\varvec{Q}}+\overline{{\varvec{Q}}}}{2}\right) , \end{aligned}$$

37 $$\begin{aligned} \overline{{\varvec{Q}}} - {\varvec{Q}}&= \hbar \frac{{\varvec{P}}+\overline{{\varvec{P}}}}{2}. \end{aligned}$$

These formulas show that $F_0 = \Phi _{\theta _0}$, which implies that $F_\gamma$ comprises a non-resonant, Hamiltonian, nearly periodic map. In particular, this map admits an all-orders adiabatic invariant $\mu = \mu _0 +\hbar \,\mu _1 + O(\hbar ^2)$, where

$$\begin{aligned} \mu _0&= \tfrac{1}{2}(q^2+p^2)\\ \mu _1&= -\frac{1}{2}W({\varvec{Q}})\bigg (2\,p\,q + (p-q)(p+q)\,\cot \theta _0\bigg ). \end{aligned}$$

Moreover, Eqs. (36) and (37) provide a consistent numerical scheme for the averaged dynamics of the slow variables. To see this, observe that the average of $q^2$ in (36) after many iterations tends to $\mu _0$, which implies that, on average, (36) and (37) comprise the implicit midpoint scheme applied to the continuous system’s averaged dynamics. Note that the relationship between the physical timestep *h* and $\hbar$ is $h = \hbar /\epsilon$. Also note that the approximation $q(h/2)\approx q(0)$ used when approximating the action integral is not systematic due to the rapid oscillations in *q*(*t*). This was done merely for the sake of obtaining an especially simple time advance. A more systematic approach would adopt Filon-type quadrature for the part of the integrand involving both slowly and rapidly varying terms, but the resulting nearly periodic map would have the same qualitative properties as the one introduced here.

A planar *N*-body problem in Cartesian (*x*, *y*)-coordinates provides a convenient sandbox for testing the novel scheme (34)–(37). Assume two bodies, labeled by the position vectors $Q_1=(Q_{1,x},Q_{1,y})$ and $Q_2=(Q_{2,x},Q_{2,y})$ and the respective momentum vectors $P_1=(P_{1,x},P_{1,y})$ and $P_2=(P_{2,x},P_{2,y})$, to orbit an infinitely massive body at the origin. The potential $V({\varvec{Q}})$ is therefore

38 $$\begin{aligned} V(Q_1,Q_2)&= -\frac{1}{|Q_1|} - \frac{1}{|Q_2|}. \end{aligned}$$

Also assume the two bodies to interact via the additional central potential

39 $$\begin{aligned} W(Q_1,Q_2)&= -\frac{1}{|Q_1-Q_2|}. \end{aligned}$$

The instantaneous value of $q^2$ therefore indicates the strength of the coupling of the two bodies via the temporal evolution of the $\epsilon$-perturbed (*q*, *p*) oscillator.

**Fig. 1:**
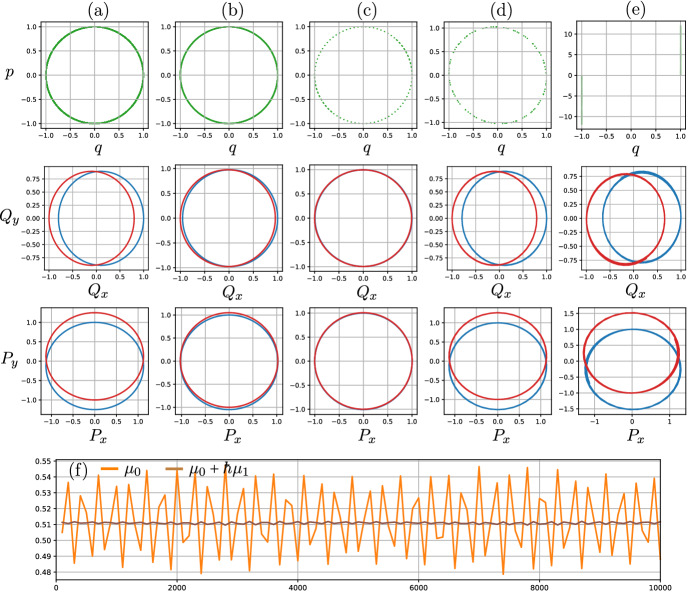
Numerical solutions of the hidden-variable Newtonian gravity example for $\epsilon =0.001$ and maximum integration time $T=10000$. The green trajectories on the top row denote the fast variable pair (*q*, *p*). The blue and red trajectories on the mid row refer to the positions $(Q_x,Q_y)$ of the particles one and two in Cartesian coordinates, and to their respective momentums $(P_x,P_y)$ on the bottom row. The columns (**a**), (**b**), and (**c**) correspond to implicit midpoint scheme with time steps $h=0.1$, $h=4.0$, and $h=100.0$, respectively. The columns (**d**) and (**e**) correspond to the fast–slow scheme with a time step of $h=100.0$ and the angle variable being (**d**) non-resonant $\theta _0=2.0$ and (e) resonant $\theta _0=\pi$. The large panel (**f**) at the bottom displays the time trace of the adiabatic invariant, corresponding to the fast–slow trajectory of column (**d**). The column (**a**) can be considered as the reference solution which the non-resonant fast–slow scheme reproduces quite well in the column (**d**)

The behavior of the scheme (34)–(37) is illustrated in Fig. 1 together with the numerical solution from the well-known implicit midpoint scheme which is symplectic for canonical Hamiltonian systems and generally considered a good scheme for stiff problems. For both integrators, we set the system parameter to $\epsilon =0.001$. In Fig. 1, the columns (a), (b), and (c) correspond to implicit midpoint scheme with time steps $h=0.1$, $h=4.0$, and $h=100.0$, respectively. The columns (d) and (e) correspond to the fast–slow scheme with a time step of $h=100.0$ and the angle variable being (d) non-resonant $\theta _0=2.0$ and (e) resonant $\theta _0=\pi$. The column (a) can be considered as the reference solution, which the non-resonant fast–slow integrator in column (d) closely matches.

Certain peculiar behavior is evident in the columns (b), (c), and (e). By increasing the time step and “stepping over” the fastest stiff time scale, the implicit midpoint method decouples the fast and slow variables but in an incorrect manner: through the columns (a), (b), and (c), the blue and red orbits gradually transit into co-centric circles, indicating the dependence of the adiabatic invariant $\mu _0$ on the step size *h*. Explanation for this behavior is rooted in the asymptotic behavior of the scheme, which is made transparent by writing the implicit midpoint scheme in the form

40 $$\begin{aligned} {\overline{q}}+q&=-2\frac{{\overline{p}}-p}{h} -\epsilon \,4\,\frac{{\overline{q}}+q}{2}\,W\left( \frac{{\varvec{Q}}+{\varvec{{\overline{Q}}}}}{2}\right) , \end{aligned}$$

41 $$\begin{aligned} {\overline{p}}+p&= 2\frac{{\overline{q}} - q}{h}, \end{aligned}$$

42 $$\begin{aligned} \overline{{\varvec{P}}} - {\varvec{P}}&= -h\epsilon \, V^\prime \left( \frac{{\varvec{Q}}+\overline{{\varvec{Q}}}}{2}\right) - h\epsilon \,\frac{({\overline{q}}+q)^2}{4}\,W^\prime \left( \frac{{\varvec{Q}}+\overline{{\varvec{Q}}}}{2}\right) , \end{aligned}$$

43 $$\begin{aligned} \overline{{\varvec{Q}}} - {\varvec{Q}}&= h\epsilon \frac{{\varvec{P}}+\overline{{\varvec{P}}}}{2}. \end{aligned}$$

When $\epsilon$ is small and the time step *h* becomes large, the limiting behavior of the fast variables is constant flipping of their signs. Most importantly, the term ${\overline{q}}+q$ becomes successively smaller with increasing *h*. Consequently, the force from the coupling potential *W* effectively drops out from the equation for the slow variables ${\varvec{P}}$, resulting in nearly co-centric slow orbits.

Also in column (e), some strange behavior occurs. Via (34) and (35), the resonant value of $\theta _0=\pi$ results in the following map for the fast variables

44 $$\begin{aligned} {\overline{p}}&= -p +\hbar \,2q\,W\left( \frac{{\varvec{Q}}+\overline{{\varvec{Q}}}}{2}\right) , \end{aligned}$$

45 $$\begin{aligned} {\overline{q}}&= -q, \end{aligned}$$

displaying a constant flipping of *q*, which translates to amplifying flipping in *p*. This behavior is straightforward to verify for the initial condition $(q,p)=(1.0, 0.0)$. The behavior of the slow variables in the column (e) in Fig. 1 is off from the reference solution and the non-resonant case but, because the dependence of (36) is only on *q* and not on the midpoint $({\overline{q}}+q)/2$, the solution still exhibits some effect from the coupling potential. Specifically, as the $q^2$ remains constant and does not average to $(q^2+p^2)/2$, the effect is actually double that of the one in the reference solution, resulting in the slow orbits being further apart from each other than what they should be.

This example serves to illustrate that even the decorated implicit midpoint scheme is not guaranteed to result in correct asymptotic behavior and that care should be taken in trying to “step over” the stiff time scales. On the other hand, the example also illustrates that an integrator with the correct asymptotic behavior may be constructed, although care is needed in choosing the saturation value for phase angle for the limit of the nearly periodic map.

### Reduced Guiding-Center Motion

We now apply the general theory developed in Sect. 4.2 to motion of a charged particle in a strong magnetic field of the special form ${\varvec{B}}(x,y,z) = B(x,y)\,{\varvec{e}}_z$, where (*x*, *y*, *z*) denotes the usual Cartesian coordinates on ${\mathbb {R}}^3$ and *B* is a positive function. Let $q=(x,y) \in Q={\mathbb {R}}^2$ and introduce a symplectic form $\omega$ on *Q* using the formula

$$\begin{aligned} \omega = -{\textbf{d}}\alpha =-B(x,y)\,dx\wedge dy,\quad \alpha = A_x(x,y)\,dx + A_y(x,y)\,dy. \end{aligned}$$

Here, the components of the 1-form $\alpha$ may be interpreted as the physicist’s vector potential for *B*. Also define the Hamiltonian function $H:Q\rightarrow {\mathbb {R}}$ according to

$$\begin{aligned} H(q) = \mu B(x,y), \end{aligned}$$

where $\mu$ is a positive constant parameter. The corresponding Hamiltonian vector field is given by

$$\begin{aligned}X_H=\,{\mathcal {R}}_{\pi /2}\,\mu \nabla \ln B,\end{aligned}$$

where ${\mathcal {R}}_{\pi /2}$ is the rotation matrix ${\mathcal {R}}_{\theta }$ evaluated at $\pi /2$,

$$\begin{aligned} {\mathcal {R}}_{\theta }=\begin{pmatrix} \cos \theta &{} -\sin \theta \\ \sin \theta &{} \cos \theta \\ \end{pmatrix}. \end{aligned}$$

Physically, this Hamiltonian vector field describes the motion of a charged particle’s guiding center (Northrop 1963) (*x*, *y*). The parameter $\mu$ is the magnetic moment, and $X_H$ is also known as the $\nabla B$-drift velocity. We remark that readers familiar with the Hamiltonian formulation of guiding center motion (Littlejohn 1981; Cary and Brizard 2009) may be used to seeing these equations derived from the Lagrangian $L:TQ\rightarrow {\mathbb {R}}$ given by

$$\begin{aligned} L(q,{\dot{q}})=\alpha _q({\dot{q}})-H(q). \end{aligned}$$

We also remark that in this formulation of guiding center dynamics we have used translation invariance along *z* to eliminate the (constant) velocity along the magnetic field and the corresponding ignorable coordinate *z*.

In order to construct the symplectic Lorentz map for this system, we begin by observing that the complex structure

$$\begin{aligned} {\mathbb {J}}\begin{pmatrix} {\dot{x}}\\ {\dot{y}} \end{pmatrix} = \begin{pmatrix} 0 &{} 1\\ -1 &{} 0 \end{pmatrix}\begin{pmatrix} {\dot{x}}\\ {\dot{y}} \end{pmatrix} \end{aligned}$$

is compatible with $\omega$ since

$$\begin{aligned} \omega ({\dot{q}}_1,{\mathbb {J}}{\dot{q}}_2) = B(x,y)\,{\dot{q}}_1\cdot {\dot{q}}_2, \end{aligned}$$

where $\cdot$ denotes the usual inner product on ${\mathbb {R}}^2$. We may therefore use the metric $g_q(v,w)=B(x,y)v\cdot w$ to build a new Hamiltonian system on *TQ*, compatible with the Lorentz-embedding idea from Burby and Hirvijoki (2021),

$$\begin{aligned} \Omega _{\epsilon }^*=\pi ^*\omega -\epsilon \,{\textbf{d}}(g_q(v,dq)), \qquad H_\epsilon ^*(q,v)=\frac{1}{2}\epsilon ^2\,g_q(v,v)+\epsilon \,\tau H(q), \end{aligned}$$

where we have also introduced a constant factor $\tau$ for scaling time of the original system. This scaling will help us later to assign the fastest motion in the guiding center system to occur at order one and help in nonlinear solve of the coordinate update map in our numerical example. Essentially, the true time of the original system now evolves at rate that is $\tau$ times the rate of the embedded system. The equations of motion for this larger system are given by

$$\begin{aligned} {\dot{q}}&=\epsilon v,\\ {\dot{v}}&=-(1+\epsilon v\cdot {\mathcal {R}}_{\pi /2}\cdot \nabla \ln B)\,{\mathcal {R}}_{\pi /2}v-\left( \tau \mu +\epsilon \frac{1}{2}|v|^2\right) \nabla \ln B. \end{aligned}$$

Proceeding now with the general construction of the symplectic Lorentz map, we introduce a Type I generating function

$$\begin{aligned} S(q,{\overline{q}})&= \int _q^{{\overline{q}}}\alpha + \Sigma (q/2+{\overline{q}}/2,{\overline{q}}-q) , \end{aligned}$$

where $\Sigma :TQ\rightarrow {\mathbb {R}}$ is given by

$$\begin{aligned} \Sigma (\eta ,\xi )&= -\hbar \mu B(\eta ) + \hbar ^2\,B(\eta )\,X_H(\eta )\cdot \xi \nonumber \\&\qquad - \frac{1}{4}\left( \frac{\sin \theta _0}{1-\cos \theta _0}\right) B(\eta )\,(\xi -\hbar X_H(\eta ))\cdot (\xi -\hbar X_H(\eta )). \end{aligned}$$

Note we are using the symbol $\eta$ instead of *x*, in contrast to Sect. 4.2, in order to avoid confusion with the standard Cartesian coordinate system. The term involving derivatives of $\omega$, present in the previous section, vanishes identically for $X_H\cdot \nabla B=0$. The symplectic Lorentz map then provides the equations

$$\begin{aligned}&\hbar ^2B({\overline{q}}){\overline{v}}=-\int _0^1\lambda B(q+\lambda \xi )d\lambda \,{\mathcal {R}}_{\pi /2}\xi +\frac{1}{2}\partial _\eta \Sigma (\eta ,\xi )+\partial _\xi \Sigma (\eta ,\xi ),\\&\hbar ^2B(q)v=\int _0^1(1-\lambda ) B(q+\lambda \xi )d\lambda \,{\mathcal {R}}_{\pi /2}\xi -\frac{1}{2}\partial _\eta \Sigma (\eta ,\xi )+\partial _\xi \Sigma (\eta ,\xi ), \end{aligned}$$

where the derivatives are

$$\begin{aligned} \partial _\eta \Sigma (\eta ,\xi )&=-\hbar \mu \nabla B(\eta ) + \hbar ^2\,\nabla B(\eta )\,X_H(\eta )\cdot \xi + \hbar ^2\,B(\eta )\,\nabla X_H(\eta )\cdot \xi \nonumber \\&\qquad - \frac{1}{4}\left( \frac{\sin \theta _0}{1-\cos \theta _0}\right) \nabla B(\eta )\,(\xi -\hbar X_H(\eta ))\cdot (\xi -\hbar X_H(\eta ))\nonumber \\&\qquad +\frac{1}{2}\left( \frac{\sin \theta _0}{1-\cos \theta _0}\right) B(\eta )\,\hbar \nabla X_H(\eta )\cdot (\xi -\hbar X_H(\eta )), \\ \partial _\xi \Sigma (\eta ,\xi )&= \hbar ^2\,B(\eta )\,X_H(\eta ) - \frac{1}{2}\left( \frac{\sin \theta _0}{1-\cos \theta _0}\right) B(\eta )\,(\xi -\hbar X_H(\eta )), \end{aligned}$$

and everything is understood to be evaluated at $(\eta ,\xi )=(({\overline{q}}+q)/2,{\overline{q}}-q)$.

Next, we rearrange the implicit equation for ${\overline{q}}$ into

$$\begin{aligned}&\left[ \frac{1}{2}\left( \frac{\sin \theta _0}{1-\cos \theta _0}\right) B(\eta )\,1-\int _0^1(1-\lambda ) B(q+\lambda \xi )d\lambda \,{\mathcal {R}}_{\pi /2}\right] \xi \\&=\left[ \frac{1}{2}\left( \frac{\sin \theta _0}{1-\cos \theta _0}\right) B(\eta )\,1-\frac{1}{2}B(\eta ){\mathcal {R}}_{\pi /2}\right] \,\hbar X_H(\eta )\\&\qquad +\hbar ^2\,B(\eta )\,X_H(\eta )-\hbar ^2B(q)v -\frac{1}{2} \hbar ^2\,\nabla B(\eta )\,X_H(\eta )\cdot \xi -\frac{1}{2} \hbar ^2\,B(\eta )\,\nabla X_H(\eta )\cdot \xi \\ {}&\qquad + \frac{1}{8}\left( \frac{\sin \theta _0}{1-\cos \theta _0}\right) \nabla B(\eta )\,(\xi -\hbar X_H(\eta ))\cdot (\xi -\hbar X_H(\eta )) \\ {}&\qquad -\frac{1}{4}\left( \frac{\sin \theta _0}{1-\cos \theta _0}\right) B(\eta )\,\hbar \nabla X_H(\eta )\cdot (\xi -\hbar X_H(\eta )), \end{aligned}$$

which can be iterated for $\eta$ and $\xi$. After that, one solves for ${\overline{v}}$ by evaluating, for example, the expression

$$\begin{aligned} \hbar ^2B({\overline{q}}){\overline{v}}+\hbar ^2B(q)v&=-\int _0^1\lambda B(q+\lambda \xi )d\lambda \,{\mathcal {R}}_{\pi /2}\xi +\int _0^1(1-\lambda ) B(q+\lambda \xi )d\lambda \,{\mathcal {R}}_{\pi /2}\xi \\&\qquad +2\hbar ^2\,B(\eta )\,X_H(\eta ) - \left( \frac{\sin \theta _0}{1-\cos \theta _0}\right) B(\eta )\,(\xi -\hbar X_H(\eta )). \end{aligned}$$

**Fig. 2:**
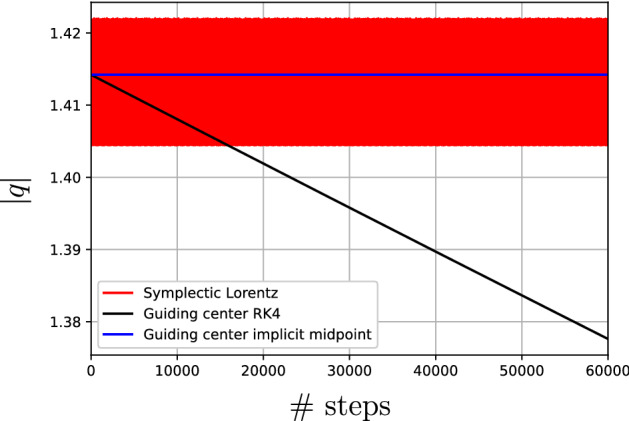
Comparison of the guiding-center RK4 integrator, implicit midpoint, and the symplectic Lorentz map in the simple magnetic field case. The orbit radius |*q*| of the RK4 integrator deteriorates clearly while the symplectic Lorentz map and the implicit midpoint manage to retain the radius within stable limits

Next, we perform some numerical tests. First, we choose a magnetic field

$$\begin{aligned} B=B_0(1+\alpha |q|^2), \end{aligned}$$

where $\alpha$ introduces a small perturbation to the otherwise constant magnetic field. For the original system ${\dot{q}}=X_H(q)$, this field results in circular orbits for *q*. We then investigate the solutions of the symplectic Lorentz map and compare them with the classic RK4 integrator and the implicit midpoint applied to the original system. For the scaling of time, we choose $\tau =\alpha ^{-1}$. Choosing an initial point $q=(1,1)$, parameters $B_0=1$, $\mu =1.0$, $\alpha =0.001$, and initializing the Lorentz map with $v=X_H(q)$, we run the simulation for 60’000 steps of size $\hbar =0.1$. This is enough to demonstrate the deterioration of the RK4 method while the symplectic Lorentz map and the implicit midpoint preserve the orbit in place, as seen in Fig. 2. The average number of iterations for solving the discrete system of equations and the average total execution times for the different algorithms are recorded in Table 1. Solving the nonlinear equations to machine precision results on average 16 iterations for the implicit midpoint and 22 iterations for the symplectic Lorentz map during every time step. The execution time of the Lorentz map is approximately six times that of the implicit midpoint method, credited to the larger number of iterations required and the need for additional quantities to be evaluated, such as the line integrals present in the generating function. This limited comparison with other methods should not be construed as the last word on the subject. The important takeaways include (a) the number of implicit iterations needed for one step of the symplectic Lorentz map is comparable to the number of iterations required for implicit midpoint applied to the original problem, even though the number of function evaluations is higher for symplectic Lorentz, (b) the symplectic Lorentz map achieves similar long-time solution quality as a popular scheme for integrating non-dissipative systems, and (c) the symplectic Lorentz map has provable structure-preserving properties, while implicit midpoint does not in this case. (Implicit midpoint is known to be symplectic for canonical Hamiltonian systems, but not in general for non-canonical systems like the one considered here.)

**Table 1:** Cost performance of different integrators

	RK4	Implicit midpoint	Symplectic Lorentz
Average # of iterations	1	16	22
Average execution time	1.4	21	128

**Fig. 3:**
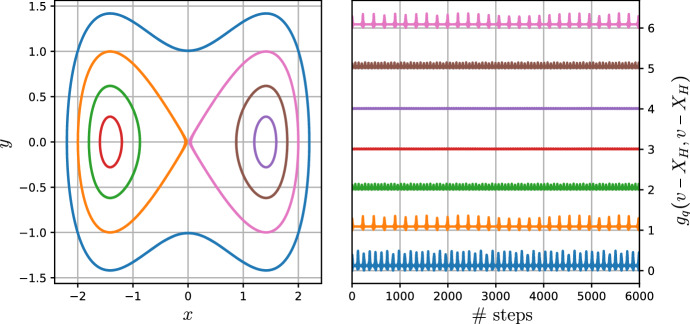
Phase-space orbits (left) and the adiabatic invariant (right). For the “figure-eight” magnetic field. The values of the adiabatic invariant have been shifted by the integers $\{0,...,6\}$ for illustrative purposes. A lesser number of steps (6000) have been chosen to illustrate the non-trivial beating structure of the adiabatic invariant

Next, we consider the magnetic field

$$\begin{aligned} B(x,y)=2+y^2-x^2+\frac{1}{4}x^4, \end{aligned}$$

whose level sets have a “figure-eight” structure. By energy conservation, the guiding-center orbits should reflect this pattern. Choosing a time step of $\hbar =0.05$ and $\tau =1.0$, we run the symplectic Lorentz map for 6’000 steps and illustrate both the orbits and the evolution of the postulated adiabatic invariant $g_q(v-X_H,v-X_H)$ in Fig. 3. The orbits appear stable and well-confined to their respective phase-space domains, and the adiabatic invariant remains within bounds while oscillating with a non-trivial beating structure.

**Fig. 4:**
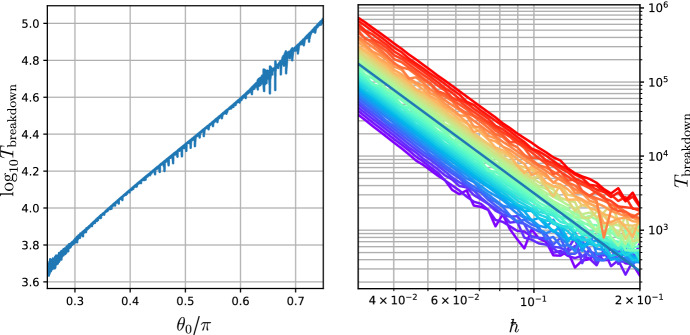
Left panel: Breakdown time for the adiabatic invariant versus $\theta _0$. Timestep is fixed at $\hbar = 0.05565$. Initial condition is $x = 2.0$, $y = 0.0$. Right panel: Breakdown time for the adiabatic invariant versus $\hbar$. Colorscale indicates value of $\theta _0$, ranging from $\theta _0 = \pi /4$ (purple) to $\theta _0 = 3\pi /4$ (red). Initial condition is $x = 2.0$, $y = 0.0$. Central dark green line is $\hbar ^{-3.5}$, for reference. Theory predicts that the breakdown time should scale like $\hbar ^{-N}$ for any positive *N* when $\hbar$ is small enough. While superpolynomial scaling of the breakdown time as a function of $\hbar$ is not apparent in these computations, it cannot be ruled out given the limited range of $\hbar$ values considered. Computing the breakdown time for appreciably smaller values of $\hbar$ rapidly becomes prohibitively expensive because the adiabatic invariant is so well conserved

For the same magnetic field, we performed a pair of tests that probe the robustness of the discrete-time adiabatic invariant $\mu$. We introduce an empirical estimate of the breakdown time for $\mu$ conservation and compute how that estimate varies with the parameters $\theta _0$ and $\hbar$. Our estimate is based on the observation that $\mu$ typically oscillates about a time-varying mean value ${\overline{\mu }}$ with an approximately constant oscillation amplitude ${\tilde{\mu }}$. We estimate that breakdown has occurred after *n* iterations when ${\overline{\mu }}(n\,\hbar )-{\overline{\mu }}(0)> {\tilde{\mu }}$. We then define the breakdown time estimate to be $T_{\text {breakdown}} = n\,\hbar$. Results from our sensitivity studies are displayed in Fig. 4. While the general theory predicts that the breakdown time should scale as fast as $\hbar ^{-N}$ for any nonnegative integer *N*, the observable asymptote in $T_{\text {breakdown}}(\hbar )$ appears well-approximated by $\hbar ^{-3.5}$. We presently lack understanding of the origin of the scaling exponent $-3.5$. The theory also predicts that adiabatic invariance should be less robust when $\theta _0/2\pi$ is rational. This prediction is consistent with the plot of $T_{\text {breakdown}}(\theta _0)$, which shows intermittent depressions in the breakdown time superposed on a strong upward trend as $\theta _0$ approaches $\pi$. We hypothesize that these depressions occur at small denominator rational values of $\theta _0/2\pi$ that produce nonlinear self-resonance in the integrator. As with the scaling exponent, we presently lack detailed understanding for the dramatic increase in observed breakdown time as $\theta _0$ approaches $\pi$.

## Discussion

In this article, we have introduced and developed the theoretical foundations of nearly periodic maps. These maps provide a discrete-time analog of Kruskal (1962) continuous-time nearly periodic systems. The limiting dynamics of both nearly periodic systems and nearly periodic maps translate points along the orbits of a principal circle bundle. In the continuous-time case, each limiting trajectory ergodically samples an orbit. In discrete time, non-resonance appears as an additional requirement for ergodic sampling. As a first major application of nearly periodic maps, we used them to construct a class of geometric integrators for Hamiltonian systems on arbitrary exact symplectic manifolds.

Kruskal’s principal interest in continuous-time nearly periodic systems came from their relationship to the theory of adiabatic invariants. In the paper (Kruskal 1962), Kruskal showed that nearly periodic systems necessarily admit approximate *U*(1)-symmetries. He then went on to deduce that this approximate symmetry implies the existence of an adiabatic invariant when the underlying nearly periodic system happens to be Hamiltonian. The theory of nearly periodic maps is satisfying in this respect since it establishes the existence of a discrete-time adiabatic invariant for nearly periodic maps with an appropriate Hamiltonian structure. Moreover, the arguments used in the existence proof parallel those originally used by Kruskal. (See Thm. 4.)

It is useful to place the integrators developed in this article in the context of previous attempts at geometric integration of non-canonical Hamiltonian systems. Based on the observation (Arnold 1989) that Hamiltonian systems on exact symplectic manifolds admit degenerate “phase space Lagrangians” (Cary and Littlejohn 1983), Qin and Guan (2008) proposed direct application of the theory of variational integration (Marsden and West 2001) to phase space Lagrangians for non-canonical systems. While initial results looked promising, further investigations by Leland Ellison (2016); Ellison et al. (2017) revealed that the most intuitive variational discretizations of phase space Lagrangians typically suffer from unphysical instabilities known as “parasitic modes” (Hairer et al. 2006). As noticed first in Rowley and Marsden (2002), the origin of these parasitic modes is related to a mismatch between the differing levels of degeneracy in the phase space Lagrangian and its discretization. Our integrators may be understood as modifications of those studied by Qin and Ellison that stabilize the parasitic modes over very large time intervals by way of a discrete-time adiabatic invariant. This “adiabatic stabilization” mechanism is conceptually interesting since it suppresses numerical instabilities without resorting to the addition of artificial dissipation. Also of note, adiabatic stabilization differs from the stabilization mechanism proposed by Ellison in Ellison et al. (2017), wherein the phase space Lagrangian is discretized so that it has the same level of degeneracy as its continuous-time counterpart. While Ellison’s “properly degenerate” discretizations apply to a very limited class of non-canonical Hamiltonian systems, (see Ellison et al. 2017 for the precise limitations) the adiabatic stabilization method discussed here applies to any Hamiltonian system on an exact symplectic manifold.

In the preprint (Kraus 2017), Kraus has developed an alternative approach to structure-preserving integration of non-canonical Hamiltonian systems based on projection methods. In contrast to our approach, this technique is designed to produce integrators that preserve the original system’s symplectic form, rather than a symplectic form on a larger space. However, there is no geometric picture for why Kraus’ method ought to have this property. In fact, Kraus finds that geometrically reasonable variants of his method are not symplectic. The structure-preserving properties of our method are easier to understand in this respect, since they follow from the standard theory of mixed-variable generating functions for symplectic maps. Both techniques warrant further investigation.

As a final remark concerning relationships between the theory developed here and previous work, it is worthwhile highlighting the technique introduced by Tao in Tao (2016) for constructing explicit symplectic integrators for non-separable Hamiltonians. The latter technique applies to canonical Hamiltonian systems with general Hamiltonian *H*(*q*, *p*). It proceeds by constructing a canonical Hamiltonian system in a space with double the dimension of the original (*q*, *p*) space, and then applying splitting methods to the larger system. Much like the symplectic Lorentz system introduced in Burby and Hirvijoki (2021), and exploited in Sect. 4.2, Tao’s larger system contains a copy of the original system as a normally elliptic invariant manifold. This suggests that Tao’s construction might be interpreted as an application of nearly periodic maps. It is a curious fact, however, that Tao’s error analysis suggests the oscillation frequency around the invariant manifold cannot be made to be arbitrarily large. This indicates nearly periodic map theory is not an appropriate tool for understanding Tao’s results. It would be interesting to investigate whether or not nearly periodic map theory can be used to sharpen Tao’s estimates.

More work is required to develop nearly periodic map machinery, in both theory and practice. The following is a list of just a few of the open theoretical questions in this area.

1. Non-resonant nearly periodic maps and nearly periodic systems admit formal *U*(1)-symmetries, and therefore formal reductions to the space of *U*(1)-orbits. Given an arbitrary continuous-time nearly periodic system, are there systematic strategies for constructing nearly periodic maps whose *U*(1)-reduction approximates the flow of the nearly periodic system’s *U*(1)-reduction? (We provide a simple example where this can be done in Sect. 5.1.) Such maps would provide a good approximation of the original system’s dynamics “on average.”
2. For a Hamiltonian system on an exact symplectic manifold *M*, the geometric integrators constructed in this article comprise symplectic mappings on *TM* that admit approximate invariant manifolds diffeomorphic to *M*. In light of this diffeomorphism, is there some sense in which our integrators possess an adiabatically invariant symplectic form on *M*? (Note that this does not obviously follow from the symplectic property on *TM*.)
3. A commonly touted benefit of symplectic integration is the long-time approximate preservation of energy. Proofs of this result rely on backward error analysis. Can similar techniques be used to prove that our geometric integrators approximately preserve the original Hamiltonian system’s energy, at least over large time intervals? Our initial numerical experiments suggest such a result is satisfied for as long as the discrete-time adiabatic invariant is well-conserved.
4. Our geometric integrators enjoy local $O(\hbar ^{5/2})$ accuracy. Are there extensions of these integrators with arbitrarily high-order accuracy?

## Data Availability

The datasets generated during and/or analyzed during the current study are available from the corresponding author upon reasonable request.
